# Practical supplements for prevention and management of migraine attacks: a narrative review

**DOI:** 10.3389/fnut.2024.1433390

**Published:** 2024-10-30

**Authors:** Zahra Hajhashemy, Sahar Golpour-Hamedani, Niloofar Eshaghian, Omid Sadeghi, Fariborz Khorvash, Gholamreza Askari

**Affiliations:** ^1^Student Research Committee, Isfahan University of Medical Sciences, Isfahan, Iran; ^2^Nutrition and Food Security Research Center, Department of Community Nutrition, School of Nutrition and Food Science, Isfahan University of Medical Sciences, Isfahan, Iran; ^3^Department of Neurology, School of Medicine, Isfahan University of Medical Sciences, Isfahan, Iran

**Keywords:** migraine, supplements, nutrient deficiency, narrative review

## Abstract

**Background:**

Migraine is one of the most debilitating neurological disorders that causes frequent attacks of headaches and affects approximately 11% of the global population. Deficient or even insufficient levels of vital nutrients would increase the severity and frequency of migraine attacks. Therefore, we aimed to examine the practical supplements for the prevention and management of migraine attacks.

**Method:**

This narrative review study was conducted by searching PubMed, ISI web of science, EMBASE, Google Scholar, and Scopus using the keywords of “dietary supplement” and “migraine” plus their MeSH terms. Original articles published in English language from their inception to July 27th, 2024, studies that investigated adult population (aged >18 years), and those assessing the impact of intended nutrient supplementation on clinical symptoms of migraine were included in the study.

**Result:**

Oxidative stress and low intake of antioxidants would be risk factors for migraine attacks by inducing inflammation. The secretion of inflammatory cytokines, such as tumor necrosis factor (TNF)-a, would lead to neuroinflammation and migraine episodes by increasing the cellular permeability and interactions. Evidence also indicated a direct association between phases of migraine attacks and calcitonin gene-related peptide (CGRP), mitochondrial disorders, monoaminergic pathway, disruption in brain energy metabolism, and higher serum levels of glutamate and homocysteine. Therefore, supplementation with nutrients involved in mitochondrial function, brain energy metabolism, and even methyl donors would relieve migraine attacks.

**Conclusion:**

Evidence indicated that supplementation with riboflavin, omega-3 fatty acids, alpha lipoic acid, magnesium, probiotics, coenzyme Q10, ginger, and caffeine would have favorable effects on migraine patients. However, more prospective studies are required to evaluate the effect of other nutrients on migraine patients.

## Introduction

One of the incapacitating common neurological diseases is migraine which manifests in frequent attacks of headache and approximately 11% of the worldwide population suffer from this disorder (1). Its onset is at 7 and 10.9 years old in boys and girls, respectively (2). Therefore, quality of life in both childhood and adulthood would be affected by migraine attacks (3, 4). This disorder is associated with other chronic diseases, such as hypertension, stroke, depression, anxiety, and cardiovascular diseases (2, 5, 6). Besides the genetic factors, environmental factors such as diet, lifestyle, obesity, and chronic stress are involved in migraine pathogenesis (7–10).

Although the leading cause of migraine is not clear, inflammation has been considered as one of the important risk factors. Such that, the secretion of inflammatory cytokines, such as tumor necrosis factor-*α* (TNFα), would lead to neuroinflammation and migraine episodes by increasing the cellular permeability and interactions (11, 12). Moreover, the other inflammatory cytokines like adhesion molecules would result in vascular dysfunction and consequently neuropathic pain (13). Evidence also indicated a direct association between phases of migraine attacks and calcitonin gene-related peptide (CGRP), mitochondrial disorders, monoaminergic pathway, magnesium deficiency, and higher serum levels of glutamate (13, 14). Additionally, based on human and experimental studies, cyclooxygenase-2 (COX-2) and inducible nitric oxide synthase (iNOS) contribute to keeping inflammation and neurogenic pain. Furthermore, hyperhomocysteinemia is involved in the etiology of migraine (15).

Although there are several pharmacological and non-pharmacological approaches for the treatment of migraines, evidence documented several side effects for these medications (16, 17). Therefore, it is recommended that natural agents should be used as adjunctive therapy for prevention and alleviating migraine attacks. In the current study, we reviewed the clinical trial studies that investigated the effects of the most important natural supplements on migraine to evaluate the efficacy of these supplements in migraine therapy.

## Methods

This narrative review study was conducted by searching PubMed, ISI Web of Science, EMBASE, Google Scholar, and Scopus using the keywords “dietary supplement” and “migraine” plus their MeSH terms. Original articles published in English language from their inception to July 27th, 2024, studies that investigated adult population (aged >18 years), and those assessing the impact of intended nutrient supplementation on clinical symptoms of migraine were included in the study. Data regarding the study population, type, and dose of supplements, intervention duration, and the main results were extracted from the studies. Supplementary Table S1 provided the main characteristics of included clinical trial and case-report studies which investigated the effect of nutrient supplements on migraine.

## Water soluble vitamins

**Figure 1:**
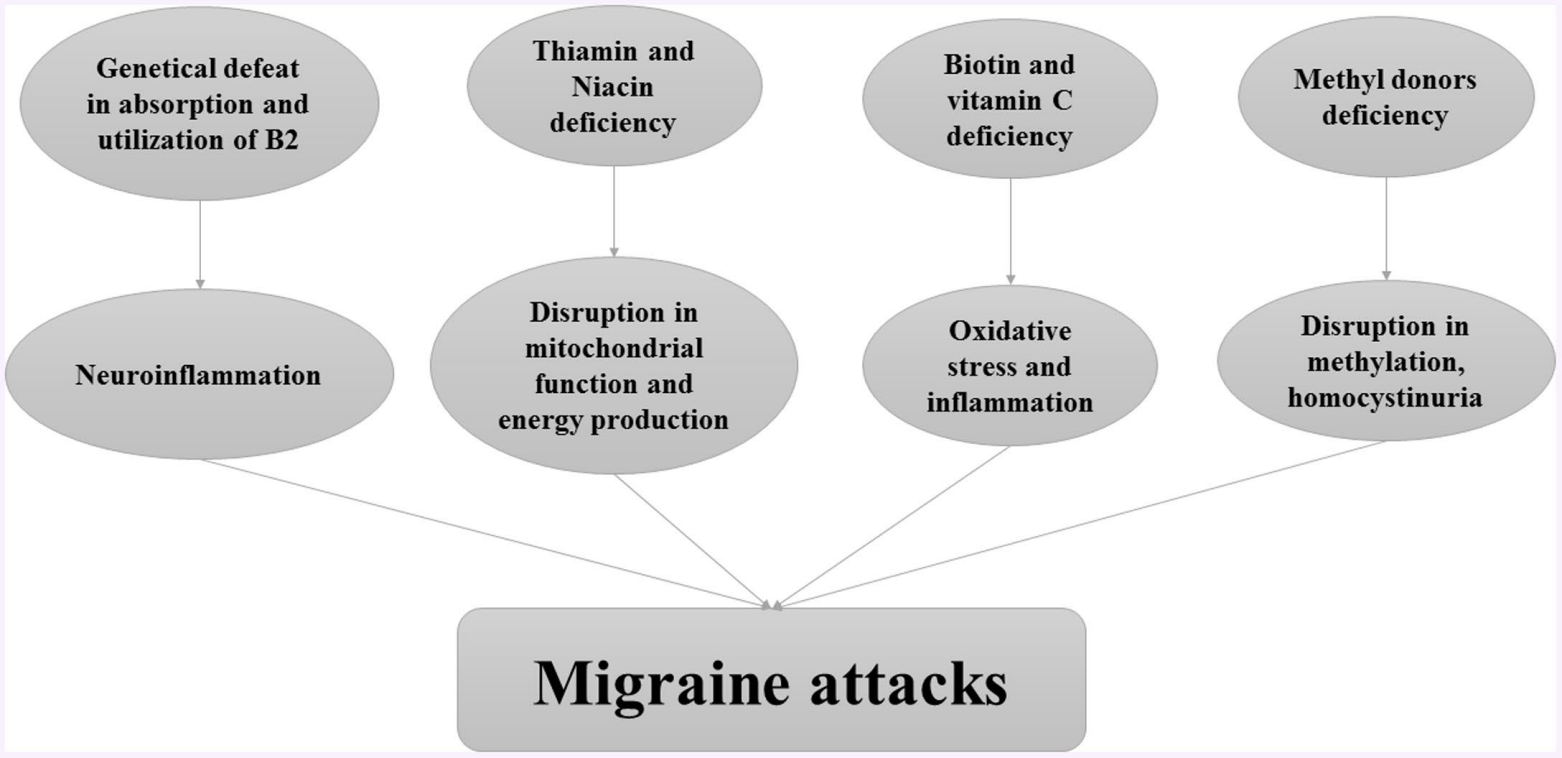
The role of water-soluble vitamins in pathogenesis of migraine attacks.

Water soluble vitamins including B complex and vitamin C, have critical roles in body metabolism. Such that, evidence indicated a positive association between water-soluble vitamin deficiency and migraine attacks. The role of water-soluble vitamins in the pathogenesis of migraine attacks is provided in Figure 1.

### Thiamine

Thiamine (vitamin B1) is one of the water-soluble vitamins that has both coenzyme and non-coenzyme roles in the metabolism of human body. It plays an important role in nerve structure and function (18). The consequences of thiamine deficiency such as increased oxidative stress, excitotoxicity, and inflammation, lead to cerebral vulnerability. In addition, cerebral inflammation is known to be a key component of some neurologic diseases including Alzheimer’s disease, multiple sclerosis, and migraine (19).

Little research has examined the relationship between thiamine status and migraine headaches. In a case study, low blood levels of thiamine due to nausea, vomiting, and anorexia were observed in two females with migraine. High dose intravenous (IV) thiamine supplementation (500 mg thiamin dissolved in 100 mL normal saline) led to a reduction in the frequency of migraine attacks (20). Another case study reported that high-dose oral thiamine supplementation (750 mg/day) improved the symptoms of chronic cluster headache, and confirmed its role in pain modulation (21).

Mitochondrial dysfunction theory is one of the hypotheses related to thiamine-migraine association (22). Thiamine has an important role in mitochondria function and is essential for the function of several enzymes in mitochondria. Despite the role of mitochondria in neural energy production, few studies investigated the effect of thiamine supplementation on migraine. Therefore, more randomized controlled trials are required in this regard.

### Riboflavin

Riboflavin is one of the most essential vitamins B due to its properties, such as anti-inflammatory, antioxidant, anti-nociceptive, and anti-aging (22–25). It is found in many food items, and its rich sources are dairy products and green vegetables. Although the riboflavin content of foods is not susceptible to cooking heat, light can decompose its structure. Evidence emphasized that a considerable worldwide population are prone to riboflavin deficiency because approximately 10–15% of people suffer from genetic defeats in the absorption and utilization of riboflavin (26). Based on the evidence, riboflavin deficiency is linked to neuroinflammation and related diseases like migraine (27).

Disruption in brain energy metabolism is considered a proposed cause of migraine attacks (28). The major pathway to supply the brain energy is oxidative metabolism, which can produce adenosine triphosphate (ATP) through an electron transport chain and reactive oxygen species (ROS) in mitochondria. Evidence indicated that the dysregulation in mitochondrial function would have negative effects on the both hemostasis of cellular ions and the stability of the cellular membrane, leading to cortex excitability(29, 30). Riboflavin is one of the important components of oxidative metabolism due to its role as the precursor to coenzymes, flavin mononucleotide (FMN), and flavin– adenosine–dinucleotide (FAD) in mitochondria (31, 32). Therefore, riboflavin deficiency might be involved in the pathogenesis of migraine (27).

A randomized clinical trial (RCT) demonstrated that high-dose riboflavin supplementation (400 mg/d) could decrease the attack frequency, duration, severity, and number of headache days in adults with migraine, after 3 months. Furthermore, the side effects in the riboflavin group were lower than those in the placebo group (33). Also, it has been indicated that high-dose riboflavin had a desirable effect on migraine prophylaxis. It was estimated that 400 mg/d riboflavin supplementation during 3–6 months could reduce the headache frequency and the use of abortive anti-migraine tablets (34). Another study also evaluated the efficacy of low-dose riboflavin in migraine headaches, compared with propranolol. They illustrated that 100 mg/d riboflavin supplementation during 3 months had a similar effect to propranolol intervention, and also riboflavin supplementation would be a safe intervention for migraine prevention (35). Moreover, another study documented that 400 mg/d riboflavin supplementation during 3 months had the same favorable effects as 500 mg/day sodium valproate on patients with migraine (36).

According to the mentioned studies, riboflavin would be a good choice for the prevention of migraine attacks. It seems that riboflavin supplementation during at least 12 weeks had migraine prophylaxis effects (34, 35). Moreover, evidence documented that riboflavin supplementation had the same therapeutic effects on patients with migraines, as compared with the common medications (35, 36). They suggested that sensitive subjects with migraine could intake riboflavin supplements. However, more prospective studies are required to define the best dose and duration for riboflavin supplementation in these patients.

### Niacin

Niacin (nicotinic acid and nicotinamide), is required for many metabolic processes. In the central nervous system, niacin is known as a key mediator of neuronal development and survival (37). Additionally, it has been established as a therapeutic option for some neurological disorders such as Parkinson’s disease, Alzheimer’s disease, and multiple sclerosis (38–40). Moreover, niacin may be effective in the management of migraine headaches. A case study reported that 375 mg of oral sustained-release niacin twice daily for 1 month, and 375 mg once daily for 2 months improved the state of a migraine patient (41). Moreover, Gedye et al. observed that a combined administration of low doses of tryptophan (500 mg), niacin (100 mg), calcium (500 mg calcium carbonate), caffeine (64 mg), and acetylsalicylic acid (ASA) (650 mg) shortly after migraine attack resulted in beneficial effects in 9 out of 12 migraine patients (42).

Current evidence has shown that a deficit of mitochondrial energy metabolism is involved in the pathogenesis of migraine headaches (43). Since niacin maintains mitochondrial energy metabolism by increasing substrate availability to complex I, it might act as an effective agent for migraine prevention (44). Taken together, some evidence suggests that niacin supplementation may have beneficial effects in migraine patients, but randomized controlled clinical trials are required to determine its clinical implications.

### Pantothenic acid

Pantothenic acid is involved in the biosynthesis of coenzyme A, cholesterol, fatty acids, and acetylcholine (45). Clinical use of pantothenic acid in certain conditions such as accelerating wound healing, lowering triglyceride levels, and improving rheumatoid arthritis symptoms is supported by previous studies (46).

Current evidence suggests that pantothenic acid deficiency causes neurological disturbances (47). It has been shown that pantothenic acid is significantly decreased in multiple brain regions in some neurological diseases such as Alzheimer’s disease and Huntington’s disease (48, 49). In addition, pantothenic acid has antioxidant and anti-inflammatory properties (50). Considering the role of oxidative stress and inflammation in migraine pathogenesis, it seems that pantothenic acid may be effective in migraine attacks. However, previous clinical trial studies did not investigate the efficacy of pantothenic acid supplementation in migraine patients. A review of case studies reported that intravenous administration of 16 mL vitamin C, 5 mL magnesium, 4 mL calcium, 2 mL B6, and 1 mL each of B12, B5, and B complex improved migraine headaches (51). Overall, it seems that pantothenic acid may be effective in migraine attacks. However, more randomized clinical trials are needed for a comprehensive conclusion.

### Pyridoxin

Vitamin B6 consists of three main forms including pyridoxine, pyridoxamine, and pyridoxal. All of these derivatives of vitamin B6 have important biological roles in the metabolism of organisms. Nevertheless, pyridoxal phosphate and pyridoxamine phosphate are the active endogenous metabolites of vitamin B6 that are involved in various biochemical reactions, including the metabolism of macronutrients, synthesis of nucleic acids, hemoglobin, and neurotransmitters (52, 53).

Previous investigations indicated that pyridoxin is involved in vascular functions. Therefore, pyridoxin supplementation would have beneficial effects on migraine patients due to the role of vascular functions in migraine attacks (54, 55). Although the mechanisms underlying this association were not exactly defined, investigations suggested some pathways to clarify the related mechanisms. They illustrated that the prevalence of mutation in the methylenetetrahydrofolate reductase (MTHFR) gene is higher in patients with migraines (56). Mutation in MTHFR C677T would lead to serum hyperhomocysteinemia by a 50% reduction in MTHFR activity (57). On the other hand, it has been illustrated that homocysteine reduction could have favorable effects on migraine disability, severity, and frequency of attacks (54). Considering that Pyridoxin is a methyl donor and is involved in the methylation pathways, it may be a good supplement to suppress the symptoms of migraine (58).

A previous RCT investigated the effect of pyridoxine or placebo on migraine patients. They illustrated that 80 mg/d pyridoxine supplementation during 12 weeks reduced the headache diary results (HDR), severity, and duration of migraine attacks, compared with the placebo (59). Two other studies also indicated that 6 months supplementation with 2 mg/d folic acid, 25 mg/d vitamin B6, and 400 mg/d B12 had favorable effects on homocysteine, disability, and severity of migraine attacks (54, 60). Considering the mentioned studies and underlying mechanisms, B6 might be a suppressor for migraine symptoms. However, more studies are required to define the optimum dose and duration for intervention with B6 in patients with migraine.

### Biotin

Biotin (or vitamin B7), as a cofactor of carboxylases, plays an important role in the metabolism of macronutrients (61). In biotin deficiency, metabolic disturbances and immune dysfunction can occur (62). Previous evidence reported a significant reduction in cerebrospinal fluid biotin in patients with multiple sclerosis and epileptics (63). In addition, it has been shown that biotin administration might be effective in some neurological diseases. For instance, a double-blind 12-month study showed administration of biotin (100 mg three times daily) reduced disability progression among individuals with progressive multiple sclerosis (64). Considering the involvement of biotin in neurological diseases, it seems that it may play a role in migraine headaches. However, both experimental studies and randomized clinical trial studies are needed to evaluate the efficacy of biotin supplementation in migraine patients.

### Folic acid

Folate is the typical name of a group of water-soluble compounds that belong to vitamin B groups and have a crucial role in deoxyribonucleic acid (DNA) biosynthesis (65). Folate is the natural form of B9 available in food items including green leafy vegetables, liver, eggs, and milk. However, the dietary intake of folate is usually lower than the recommendation of national health authorities (66). Folic acid is the synthetic form of folate and its supplement is given as dihydrofolate (DHF). Folate and folic acid are converted to tetrahydrofolic acid (THF) in the body to contribute to methylation pathways (67).

The low dietary folate intakes in patients with migraine could lead to disruption in mitochondrial function, energy production, and antioxidant defense, the key risk factors of migraine attacks incidence (68). However, enough folate intake could regulate these pathways by changing the DNA methylation pattern through the related mechanisms of valproic acid (VPA) (69–71). VPA is one of the important medications for migraine that could change the DNA methylation pattern through the inhabitation of the histone deacetylase and gamma-aminobutyric acid (GABA)-degrading enzymes (70). On the other hand, hyperhomocysteinemia, one of the risk factors of migraine attacks, could be regulated by methyl donors including B6, B9, and B12 (58).

An RCT investigated the effect of 5 mg/d folic acid, the combination of 5 mg/d folic acid and 80 mg/d pyridoxin, and placebo for 3 months. Although the combination of folic acid and pyridoxin had favorable effects on the HDRs, severity, frequency, and duration of migraine attacks; folic acid alone could not influence the migraine symptoms in these patients (72). Another RCT examined the effect of B1 (300 mg/d), B6 (80 mg/d), B9 (2mg/d), B12 (500 μg/d) supplements and B-complex for 12 weeks on patients with migraine, compared with the placebo. Interestingly, all of the supplements had reduction effects on the use of abortive drugs, frequency and disability of migraine attacks (73). It seems that the efficacy of B9 supplementation is dose-dependent such that, the combination of 1 mg/d folic acid, 25 mg/d vitamin B6, and 400 μg/d B12 during 6 months did not have any significant effect on serum homocysteine and symptoms of migraine (74). Nevertheless, supplementation with 2 mg/d folic acid, 25 mg/d vitamin B6, and 400 μg/d B12 during 6 months significantly decreased the homocysteine levels, severity, and disability of migraine attacks (60). Regarding the mentioned studies, it seems that a higher dosage of folic acid especially in combination with B6 and B12 could alleviate the symptoms of migraine. However, more studies are required to exactly define the best dose and duration of intervention.

### Cobalamin

B12 is named cobalamin due to its structure which contains a cobalt atom in the center of a tetrapyrrolic ring. Animal foods such as meat, eggs, and dairy products are rich sources of B12 because only bacteria can synthesize B12. As the liver storage of B12 is about 2–5 mg cobalamin, B12 deficiency (liver cobalamin <300 μg/d) would take place after several years of insufficient intake (75). Cobalamin is essential for several metabolic pathways including cell proliferation and survival, energy production, and nervous system integrity (76).

As nitric oxide (NO) is a leading cause of spontaneous migraine attacks, several hours after a NO challenge, the early phase of migraine attacks would be incident in patients. Therefore, medications with properties of NO-synthase inhibitors, endothelial receptor blockers, and NO-scavengers would be effective in relieving acute migraine attacks or even preventing them (77). Hydroxocobalamin (OHB12), one of the metabolites of cobalamin, seems to act such a NO-scavenger and might be effective in the alleviation of migraine attacks (78). Considering the low bioavailability of oral formulations of OHB12 (79), intranasal and intramuscular administration of OHB12 are alternative routes (80–82). On the other hand, considering the role of hyperhomocysteinemia in the pathogenesis of migraine attacks (15), cobalamin might be effective for migraine patients through the regulation of methylation pathways (58).

An open-label pilot study investigated the effect of 1 mg/d intranasal OHB12 administration during 3 months on 19 patients with a history of migraine >1 year. Their results indicated a significant reduction in the frequency and duration of the migraine attacks, migraine days per month, and the amount of acute migraine medication (83). Based on another RCT, a single dose of B12 (500 μg/d) or its combination with B1(300 mg/d), B6 (80 mg/d), and B9 (2mg/d) during 12 weeks had favorable effects on the frequency of headache attacks and migraine disability (73). Moreover, other studies indicated that a combination of 400 μg/d B12 with 2 mg/d folic acid and 25 mg/d vitamin B6 for 6 months, resulted in reduced homocysteine levels, severity, and disability of migraine attacks (54, 60). Taken together, it seems that B12 supplementation would be effective for migraine attack alleviation; however, more studies are required to define the optimum dose and period of intervention.

### Vitamin C

Vitamin C is a water-soluble vitamin with many biological functions in the body (84). It plays an important role in the nervous system and participates in antioxidant defense in the brain (85). It also is involved in many non-oxidant processes such as the biosynthesis of collagen, carnitine, and myelin. Furthermore, vitamin C plays an important role in neurotransmission and neuronal function (86).

The issue of vitamin C deficiency and supplementation with vitamin C in neurological diseases has been investigated in previous studies. For instance, previous evidence suggested that patients with multiple sclerosis have significantly lower levels of vitamin C compared to healthy subjects (87, 88). A clinical trial study reported that receiving 200 mg/day of vitamin C for 6 months led to the improvement of symptoms in patients with Parkinson’s disease (89). In addition, vitamin C might play a role in migraine headaches, but the efficacy of vitamin C supplementation in the prophylactic treatment of migraine has not been investigated in a randomized controlled trial. However, an RCT on 35 patients with migraine, showed that supplementation with N-acetylcysteine (1,200 mg), vitamin E (500 IU), and vitamin C (1,000 mg) daily for 3 months resulted in lower frequency, severity, and duration of headaches and lower acute medication use (90). A small uncontrolled open-label trial reported that receiving 120 mg pine bark extract, 60 mg vitamin C, and 30 IU vitamin E daily for 3 months in migraine patients led to a significant reduction in the headache frequency, severity, and migraine disability (91). In another uncontrolled open-label clinical trial, fifty patients with chronic migraine were treated with an antioxidant formulation of 1,200 mg *Pinus radiata* bark extract and 150 mg vitamin C daily for 3 months. The frequency, severity and disability of headache were decreased after 3 months. Moreover, patients who continuously took *Pinus radiata* bark extract and vitamin C combination for 12 months had more than a 50% reduction in the frequency and severity of headaches (92).

Epidemiological studies have shown an increased risk of developing complex regional pain syndrome (CRPS) among migraine patients (93). Vitamin C is thought to scavenge reactive oxygen species (ROS) produced in the early stages of CRPS (94). According to this model, vitamin C may modulate the effects of neurogenic inflammation and ROS in migraine and therefore acts as a prophylactic agent against migraine. However, more randomized clinical trials that investigate the efficacy of vitamin C supplementation in migraine attacks are needed.

### Fat-soluble vitamins

**Figure 2:**
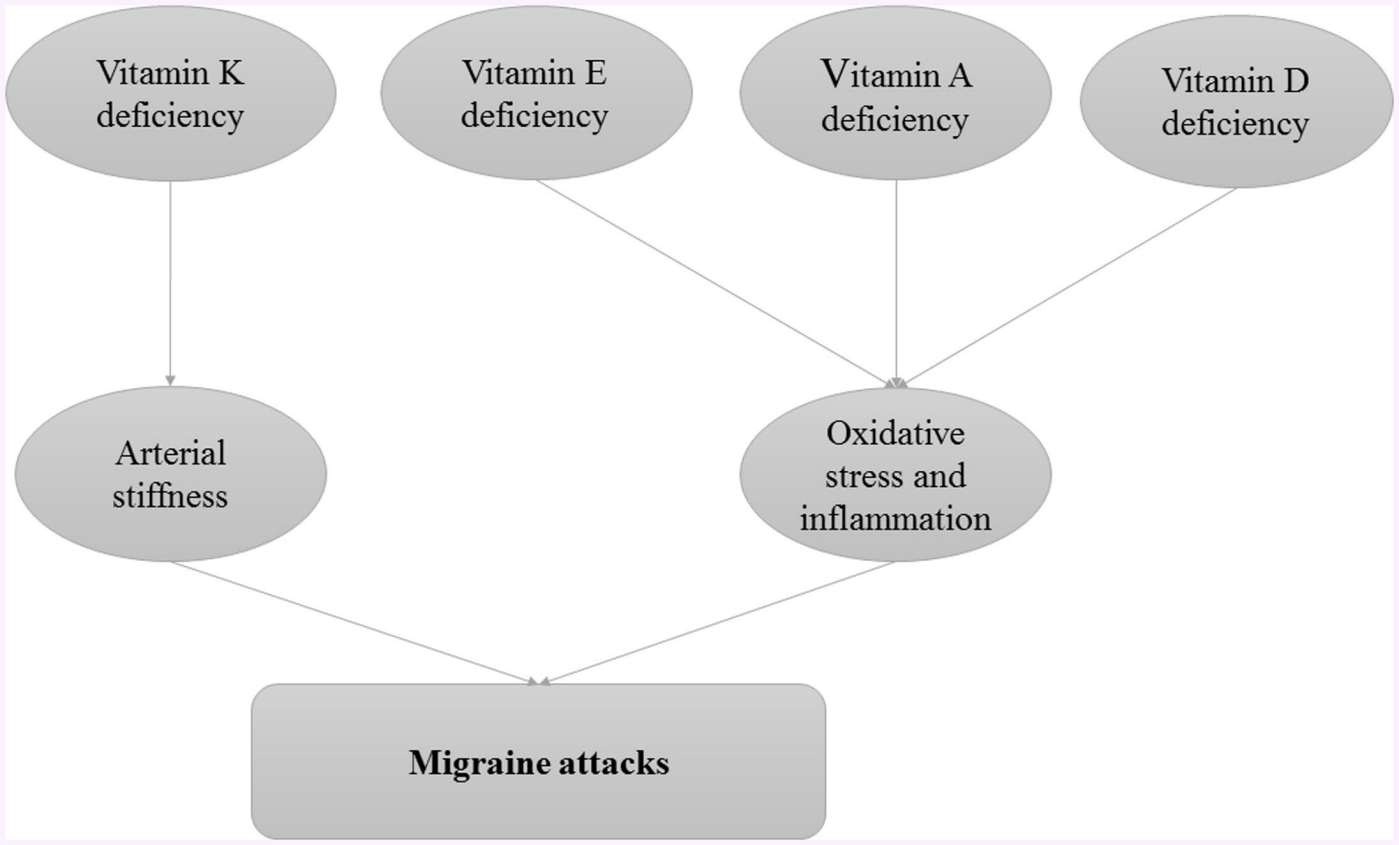
The role of fat-soluble vitamins in pathogenesis of migraine attacks.

Fat-soluble vitamins, vitamins A, D, E, and K, play crucial roles in various physiological processes, including immune function, inflammation regulation, and neuronal health (95). Investigating their impact on migraines could reveal potential therapeutic benefits or dietary interventions that could alleviate symptoms or prevent migraines. The role of fat-soluble vitamins in pathogenesis of migraine attacks is provided in Figure 2.

### Vitamin K

Vitamin K is a bioactive compound that is essential for optimal body function. Vitamin K is available in different isoforms. It has two main structures: phylloquinone (K1) and menaquinone (K2). Vitamin K2, also known as MK-7, can be considered a bioactive compound and is involved in cell survival, chemotaxis, mitogenesis, cell growth, and myelination through the activation of vitamin K-dependent proteins and sphingolipid synthesis. Vitamin K is mainly present in the brain in the form of MK-4 (96).

The beneficial effect of vitamin K supplementation on patients with thromboembolic diseases taking anticoagulant medications has been confirmed by many researchers. Anticoagulants act as vitamin K antagonists and prevent the synthesis of some clotting factors. Therefore, the intake of vitamin K supplement is particularly important in patients requiring anticoagulants (96, 97).

Mansour et al. (98) showed that patients with migraine had higher average arterial stiffness (pulse wave velocity between carotid and femur) and dephosphorylated-uncarboxylated glial protein matrix. These two indicators indicate the status of vitamin K2 in these patients. Increased arterial stiffness was associated with increased markers of vitamin K2 deficiency in the migraine with aura group. Vitamin K2 deficiency also occurred more frequently in these patients than in controls, but this association was not statistically significant (98). Specific clinical studies on the association of vitamin K with migraine are limited. However, several studies have shown the positive effects of vitamin K on various diseases of the nervous system. The protective effect of K2 on neurons has been confirmed in laboratory studies. The P38 mitogen-activated protein (MAP) kinase pathway has been proposed as a protective mechanism of vitamin K2 on neurons (99). K2 deficiency is associated with nerve spasms and optic nerve damage. Nerve spasms are also involved in the pathophysiology of migraine (100).

Due to the lack of adequate and appropriate clinical studies, there is no definitive recommendation for prescribing vitamin K to migraine patients. However, vitamin K may be useful in migraine management in patients who require anticoagulants.

### Vitamin D

Vitamin D deficiency as a global health problem affects approximately 30% to 80% of children and adults worldwide (101). The multi-step biological process of vitamin D metabolism leads to the production of cholesterol. Calcitriol is the biologically active form of vitamin D that binds to the nuclear vitamin D receptor (nVDR). Calcitriol is found in the kidneys (to increase calcium reabsorption from the glomerular filter), small intestine (to stimulate calcium absorption), and other tissues. The role of vitamin D in some brain functions is well known. Calcitriol can affect neuroplasticity, apoptosis, and gene expression (such as neurotransmitters, neurotrophic, and synaptic proteins) (102). Vitamin D, 1-alpha-hydroxylase (vitamin D-binding protein), and VDR are found in various areas of the central nervous system. Vitamin D leads to modulation of cellular oxidative stress levels, intracellular calcium concentration, immune system function, and neurotrophic factor production in the CNS. Vitamin D also inhibits the destruction mechanisms of nerve cells. Therefore, the protective role of vitamin D in protecting against neurodegenerative diseases in humans may be justified (103).

In a study, Rapisarda et al. (104) examined the association between serum vitamin D levels and migraines in 171 patients. The results showed that patients with headaches had severe vitamin D deficiency (25-hydroxyvitamin D levels below 20 ng/mL) compared to healthy people. Additionally, there was a negative linear relationship between the number of days with headache and serum vitamin D levels in patients with migraine (104). Mottaghi et al. (105) also examined the effect of vitamin D supplementation (50,000 units/week) on migraine symptoms in 65 patients with migraine, divided into control and intervention groups, over a period of 10 weeks. The results showed a significant reduction in the migraine frequency, and headache diary result in the intervention group compared with control group (105). Niu et al. (106) examined the association between vitamin D levels and migraine using a two-sample mendelian randomization (MR) method. They concluded that increased levels of circulating vitamin D were significantly associated with a reduced risk of migraines (106). Additionally, vitamin D intake (2000 IU/d) for 12 weeks had favorite effects on the frequency, duration, and values of glutamate and NOD-Like Receptor Protein 3 (NLRP3) in patients with chronic migraine (107). Ghorbani et al. (108) examined the effect of administering vitamin D (50 micrograms daily for 12 weeks) on migraines and their symptoms. In the study, the frequency and duration of attacks, the severity of headaches, and monthly medication consumption were significantly reduced in migraine patients compared to the placebo group. Additionally, serum levels of *iNOS* and *interleukin 6* (IL-6) were significantly reduced in the group receiving the supplement (108).

To confirm the results, Hu et al. (109) conducted a meta-analysis of clinical trials. The results showed that vitamin D supplementation could significantly reduce headache attacks per month, headache days per month, and migraine disability assessment (MIDAS) score in migraine patients. However, there was no noticeable effect on the duration of the migraine attack or the intensity of the headache (109). Ghorbani et al. (110) reported that %45–%100 of patients with migraine and headache had vitamin D deficiency, and taking vitamin D supplements (1000–4,000 IU/day) could also effectively reduce the frequency of migraine attacks.

As a result, taking nutritional supplements can improve the general condition of patients with vitamin D deficiency. However, studies on the effect of taking vitamin D supplements on migraine-related parameters, including intensity and duration, have been reported differently. Therefore, further studies are required in this regrade.

### Vitamin E

Vitamin E influences the enzymes phospholipase A2 and cyclooxygenase. Thus, it prevents the release of arachidonic acid and its conversion to prostaglandins (PGs) (111). The study by Armagan et al. (112) showed that vitamin E levels were lower in migraine patients without an active attack phase than in healthy people. They also reported that these patients had higher levels of *interleukin 1* (IL-1), IL-6, and TNF-*α*, suggesting an inflammatory state and oxidative stress in migraine patients (112). Vitamin E could play an important role in removing ROS. Vitamin E is the main antioxidant in the lipid phase of cells and removes lipid hydroxyl radicals in the lipid phase of cells. The results of the laboratory study by Nazroglu et al. (113) also confirm a significant decrease in vitamin E levels in rat suffering from migraine damage (113).

Ziaei et al. (114) reported that pain severity, functional disability scales, photophobia, phonophobia, and nausea were significantly reduced in patients with menstrual migraine after taking vitamin E supplements (400 units daily for 5 days, starting 2 days before to 3 days after menstruation for two cycles) (114). Several mechanisms have been proposed for menstrual migraine, including reduced magnesium levels, platelet dysfunction, impaired central serotonin modulation, and impaired prostaglandin release (115). According to study results, vitamin E has positive effects on migraines and related diseases through its antioxidant function. However, more studies are warranted to determine the exact effect of vitamin E on migraines.

### Vitamin A

Inflammation is a crucial factor in many diseases. Vitamin A (retinol) is important for the function of the immune system due to its anti-inflammatory effects. Vitamin A reduces the production of inflammatory mediators such as IL-6 and interferon 8 (IFN-8) from T cells. It also leads to an increase in anti-inflammatory mediators such as IL-4 (116, 117). Retinol-binding protein (RBP) is a plasma protein, and transports vitamin A to peripheral tissues after synthesis in liver cells and fat cells (118). Plasma RBP levels can be used to predict vitamin A deficiency. Therefore, RBP levels are low in vitamin A deficiency (119).

Tanik et al. (120) examined the association between retinol-binding protein-4 (RBP4) and high-sensitivity C-reactive protein (hs-CRP) with migraine. The results showed that the serum level of RBP4 was significantly lower in migraine patients and the hs-CRP level was also significantly higher in these patients than in controls (120). Nazroglu et al. (113) also demonstrated that a significant decrease in vitamin A was observed in rat suffering from migraine damage.

According to studies, severe oxidative stress occurs in migraine and headache. Therefore, antioxidants may be effective in both prevention and treatment of migraines (121, 122). On the other hand, vitamins A and E are very important among antioxidants. Studies confirm the positive antioxidant effects of these two vitamins. Additionally, deficiencies in fat-soluble vitamins have been observed in many patients with migraines. The National Health and Nutrition Survey (NHANES) also showed a direct connection between the prognostic nutritional index and the migraine prevalence. Of all types of fat-soluble vitamins, migraine sufferers received lower amounts of vitamins A and K per day (123). However, studies on vitamins K and A were very limited and there were very few available studies.

In summary, studying the effects of fat-soluble vitamins on migraines is crucial to better understanding the triggers of the condition and possible treatments. Such studies may contribute to more effective, personalized, and holistic approaches to managing and preventing migraines, ultimately improving the quality of life for individuals affected by this debilitating condition.

## Antioxidants

**Figure 3:**
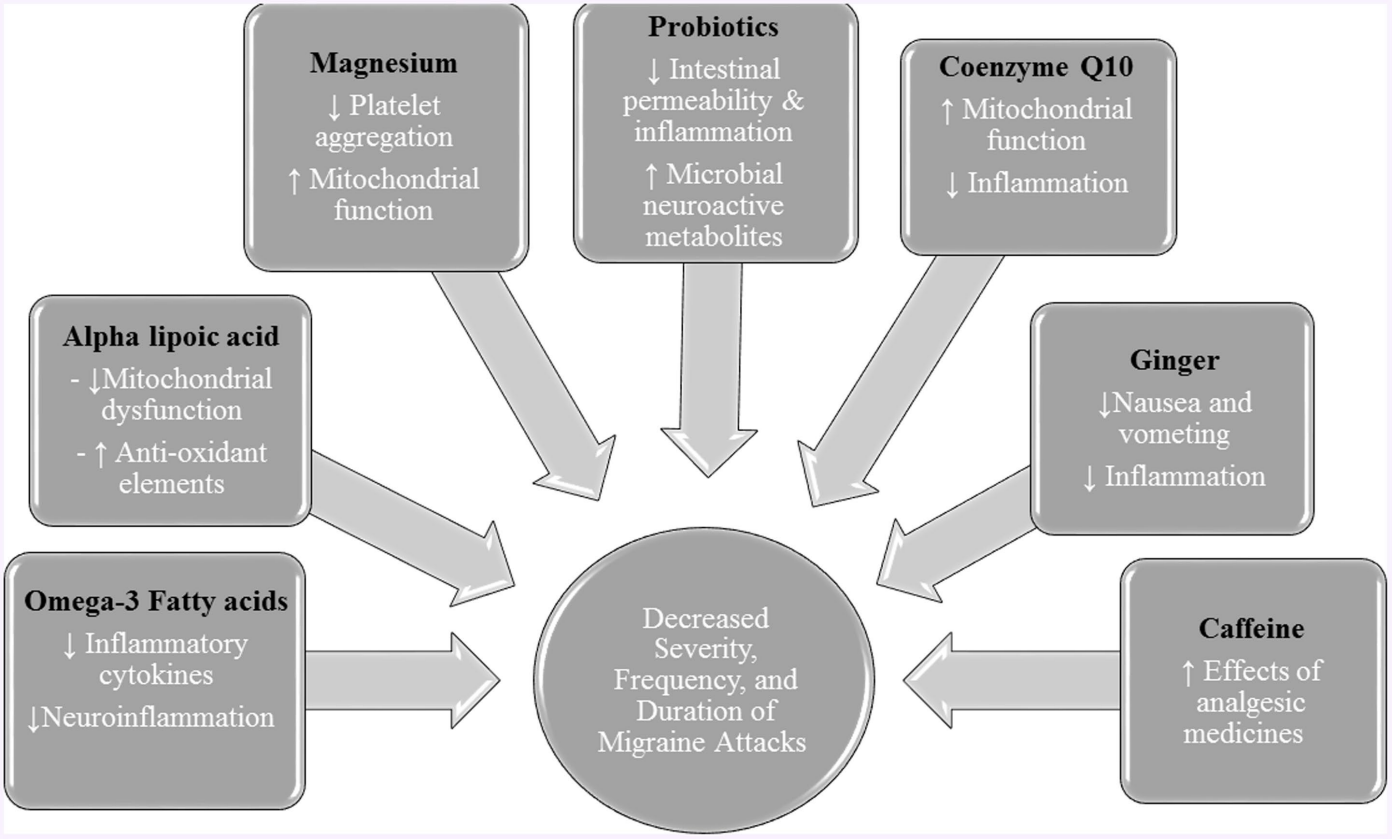
The role of antioxidants and other nutrients in alleviating migraine attacks.

Considering the role of oxidative stress and antioxidants in the pathogenesis of migraine attacks, antioxidants could alleviate the migraine attacks. Figure 3 provided the role of antioxidants and other nutrients in alleviating migraine attacks.

### Omega-3 fatty acids

Among the nutritional intakes, fatty acids (FAs) have a crucial role in determining the composition of lipid-content cell membranes and even cell signaling molecules (124, 125). The most important FAs in the brain and nerve system are omega-3 polyunsaturated fatty acids (PUFAs), such as eicosapentaenoic acid (EPA) and docosahexaenoic acid (DHA). Use of rich sources of EPA and DHA like fish oil would increase the PUFA content of cell membranes, especially immune cells involved in inflammatory responses, such as neutrophils, lymphocytes, and monocytes (126–128). Besides the anti-inflammatory effects, omega-3 fatty acids are also involved in transcription factor activity, intracellular signaling pathways, and gene expression (127, 129).

Based on the evidence, fish oil supplementation has favorable effects on autoimmune and inflammatory diseases including lupus erythematosus, psoriasis, inflammatory bowel diseases (IBD), rheumatoid arthritis, and even neuroinflammatory diseases, such as multiple sclerosis (MS) and migraine (127, 130). PUFA has neuroprotective effects by suppressing the production of NO and ROS in active microglia (131). Moreover, omega-3 fatty acids have anti-inflammatory effects on microglial cells because the resolving, one of the metabolites of omega-3 fatty acids, inhibits the production of inflammatory cytokines. Additionally, DHA could suppress the activity of nuclear factor-κB (NF-κB) through the lipopolysaccharides (LPS) surface receptor (132).

An RCT that investigated the effect of 6 g/d PUFA supplementation during 4 months on patients with migraine, found that omega-3 fatty acids did not have any significant influence on mean duration and intensity of the migraine attacks, and rescue medication use. However, the total number of attacks was decreased in intervention group (133). Another RCT that investigated the effect of 180 mg/d fish oil on migraine patients during 12 weeks, illustrated that the combination of sodium valuate and fish oil would have more favorable effects on migraine attacks, compared with the sodium valuate (134). Additionally, a high dosage of EPA (1.8 g/d) for 12 weeks could improve the psychological symptoms, and quality of life by alleviating the severity, frequency and disability of migraine attacks (135). Moreover, another study indicated that the following a diet with high omega-3 and low omega-6 fatty acids in comparison to a diet with low omega-6 had desirable effects on pain, quality of life, and antinociceptive lipid mediators in migraine patients (136). Additionally, a meta-analysis confirmed the efficacy of omega-3 supplementation on the duration of the migraine attacks (137). Furthermore, a RCT documented the anti-inflammatory effects of moega-3 supplementation in patients with migraine (138). Although the previous studies indicated the therapeutic effects of migraine supplementation, more RCT studies are required to define the best dose and duration of omega-3 supplementation for migraine patients.

### Alpha lipoic acid

Alpha-lipoic acid (*α*-LA) is an organosulfur and biological antioxidant synthesized in the mitochondria of plants, animals, and humans. This antioxidant creates covalent bonds with proteins and is involved in the Krebs cycle. Also, it acts as a cofactor in enzyme complexes (139). In recent years, α-LA has become one of the essential elements of multivitamin supplements for the prevention and treatment of many inflammatory diseases. This element has known effects on diabetes and cardiovascular disorders through its impact on inflammatory reactions (140).

Migraine is the most common neurovascular disorder, and several hypotheses have been proposed for the pathogenesis of migraine. Oxidant-antioxidant balance disorders are the most important mechanisms known in the pathogenesis of this disease (141). Increasing both levels of ROS and reactive nitrogen species (RNS) through the destruction of cellular structures, such as DNA, proteins, and lipids, can increase the risk of migraine. Many studies have confirmed oxidant-antioxidant imbalance in migraine, including NO, malondialdehyde (MDA), thiobarbituric acid reactive substances, thiol, catalase, superoxide dismutase, and glutathione imbalances (141, 142). Mitochondrial dysfunction is also considered in migraine patients (143). In this disease, the brain needs high energy levels and depends on mitochondrial function. In Addition to energy production, mitochondria also produce ROS (143, 144). *α*-LA has a high potential in eliminating oxygen and nitrogen free radicals, and regenerating various antioxidant elements, such as vitamins C and E (145).

In this regard, Eren et al. (146) investigated the antioxidant indicator in migraine patients and found that the thiol level in migraine patients was significantly lower than that in healthy people. There was a negative correlation between migraine disability score and thiol serum level in these patients. Therefore, they recommended that more studies should be conducted to investigate the effectiveness of new therapeutic approaches based on thiol-containing compounds, such as alpha-lipoic acid and N-acetylcysteine (146). In confirmation of this finding, Gross et al. (147) investigated the status of mitochondrial function markers and oxidative stress in migraine patients. The study showed that migraine patients had abnormally low serum levels of *α*-LA (87.5% of patients) and lactates (78.1% of patients). Therefore, *α*-LA is an essential biomarker for both prevention and treatment of migraine (147).

Rezaei et al. (148) investigated the effects of α-LA supplementation on migraine through a clinical trial. This study demonstrated that α-LA supplementation (300 mg/d, twice a day) for 3 months significantly decreased the serum levels of MDA and C-reactive protein (CRP), as two inflammatory and oxidative stress markers, and mood disorders in patients with episodic migraine. Therefore, *α*-LA supplementation can reduce oxidative stress and inflammation in migraine patients (148). Cavestro et al. (149) investigated the effect of α-LA supplementation (400 mg for 6 months) on headaches in patients with migraine and insulin resistance. In this study, the number of days of attack and days with treatment significantly decreased after 2, 4, and 6 months of taking the supplement (149).

Magis et al. (150) also investigated the effectiveness of *α*-LA supplementation in preventing migraine attacks. The results of the study indicated a significant decrease in the attack frequency, number of headache days, and headache severity in the group treated with the supplement (600 mg for 3 months) (150). Fogacci et al. (151) conducted a meta-analysis of clinical studies and showed that receiving *α*-LA supplements did not have any side effects, and also the use of this supplement was entirely safe and secure.

Some studies showed that the available clinical studies were small, and the samples were limited. Also, no investigation was performed on serum markers to estimate the mechanism of the supplement effect. Clinical trial studies regarding the effect of *α*-LA supplementation on migraine were limited, and the existing studies prescribed different therapeutic doses without examining the serum level of α-LA. Therefore, determining the appropriate dose and the mechanism of the supplement effect requires more studies. Finally, according to the available studies, the use of α-LA supplements can be effective in the prevention and treatment of mood disorders related to migraine. However, this issue requires more clinical studies to determine the appropriate dose and confirm its effectiveness in treating this disease.

### Coenzyme Q10

Coenzyme Q10 (CoQ10) is a natural antioxidant that plays an important role in the electron transport chain of mitochondria (152). CoQ10 is prescribed orally or parenterally and used in the treatment of many diseases, including fibromyalgia, cardiovascular disease, and some neurological disorders such as Parkinson’s disease, and Alzheimer’s disease (153, 154). Also, it may be effective in preventing migraine attacks.

The mitochondrial hypothesis of migraine has been supported by previous evidence (155, 156). Biochemical evidence indicated that energy failure due to defects in oxidative phosphorylation altered vascular tone and prevented the recycling of reactive oxygen species, which are possible mechanisms responsible for triggering migraine headaches (141). Previous studies have proven that CoQ10 produces mitochondrial-enhancing effects (157). Also, migraine has been associated with vascular inflammation (158). Previous evidence has indicated that calcitonin gene-related peptide (CGRP) and cytokines might play a role in the pathogenesis of migraine (159–161). Furthermore, it has been shown that CoQ10 has anti-inflammatory effects and is negatively associated with inflammatory markers (162). According to these findings, the role of supplementation with CoQ10 has been studied among patients with migraine.

A randomized double-blind placebo-controlled clinical trial, conducted among migraine patients, showed that CoQ10 supplementation at a dose of 400 mg/day for 3 months resulted in lower severity, duration, and frequency of headaches. In addition, lower levels of tumor necrosis factor-*α* (TNF-α) and CGRP in the serum of patients enrolled in the CoQ10-arm, suggest that these effects are through the reduction of inflammatory processes (163). In another clinical trial, Shoeibi et al. reported that receiving 100 mg/day CoQ10 for 3 months in migraine patients led to a significant reduction in the severity, duration, and frequency of migraine attacks (164). Additionally, in another RCT, the values of MDA, body fat percent (BFP), and high-density lipoprotein-cholesterol (HDL-c) were improved in migraine patients who received 400 mg/d Q10 through a 12-week period compared with the placebo group (165). Moreover, a randomized double-blind placebo-controlled clinical trial indicated that supplementation with Coenzyme Q10 (30 mg/day) and L-carnitine (500 mg/day) for 8 weeks had beneficial effects on migraine symptoms and serum levels of lactate (166). Such findings have been confirmed by a systematic review and meta-analysis in which 6 randomized clinical trials were included in the analysis (167). In addition, another meta-analysis of 4 randomized clinical trials showed that CoQ10 supplementation might reduce the frequency of migraine attacks (168). However, a study illustrated that the favorable effects of CoQ10 (400 mg/d) on migraine patients will be enhanced when used as an adjunct therapy in combination with other drugs, compared with using CoQ10 as monotherapy (169). Overall, supplementation with CoQ10 has some evidence of efficacy. Further clinical trials should be conducted to determine the beneficial effects of CoQ10 supplementation on migraine attacks.

### Ginger

Ginger (*Zingiber Officinale* Roscoe) is a pungent spice consumed worldwide, especially in South Asian countries. Ginger has several chemical compounds, including phenolic compounds, organic acids, terpenes, polysaccharides, and lipids (170). Previous studies have investigated the potential benefits of ginger extract for a wide range of chronic diseases (171, 172). Ginger has been shown to reduce pain and nausea (173). In addition, ginger may be useful in treating migraine attacks.

During migraine attacks, trigeminal nerve fibers are activated. These nerve fibers release neuropeptides that trigger the production of inflammatory mediators such as cytokines, and prostaglandins, which activate nociceptive pathways (174). Based on previous evidence, ginger components, especially gingerols, and shogaols, are able to reduce the production of prostaglandins via the inhibition of cyclooxygenase-2 (COX-2) (175). In addition, 6-shogaol plays a role in modulating neuroinflammation by inhibiting the expression of proinflammatory cytokines in microglial cells (176).

Previous studies have shown that ginger might play a role in migraine treatment. For instance, in a double-blinded randomized clinical trial, Maghbooli et al. compared the efficacy of the administration of ginger powder (250 mg) with sumatriptan (50 mg) in the treatment of migraine. Two hours after dosing, the severity of headaches decreased in both groups. However, the side effects of ginger powder were less than sumatriptan (177). In another RCT, patients were divided into two groups, in which they received ginger extract (400 mg) or placebo, in addition to an intravenous drug (100 mg ketoprofen). Patients who received ginger extract showed a better clinical response after 1, 1.5, and 2 h (178). In addition, a meta-analysis of three randomized controlled trials reported that ginger administration in migraine patients was effective on pain outcomes assessed at 2 h. Also, ginger reduced the risk of migraine-related nausea and vomiting (179).

Taken together, ginger may be effective in treating migraine attacks. Further clinical trials are needed for a more precise conclusion.

## Other effective supplements

### Magnesium

Magnesium is the fourth most abundant cation in the human body, which can play an important role in several vital body functions, such as enzyme activity, deoxyribonucleic acid (DNA) and protein synthesis, oxidative phosphorylation, and parathyroid hormone secretion (180, 181). The serum concentration of magnesium is regulated by establishing a balance between intestinal absorption, renal excretion, and bone buffer. Insufficient intake of magnesium, loss of magnesium through the digestive system and kidneys, and redistribution of magnesium from the extracellular space to the intracellular space are the main causes of magnesium deficiency (182). Acute magnesium deficiency is usually asymptomatic, and if it is symptomatic, it is associated with symptoms, such as nausea, vomiting, lethargy, mood, and nervous disorders (depression, anxiety, and stress) (183, 184). Chronic magnesium deficiency leads to widespread cardiovascular, neurological, and muscular problems (185).

Magnesium is one of the essential minerals in the functioning of nerves, whose main function maintain the electrical potential of neurons. Several studies showed the relationship between magnesium deficiency and migraine headaches (186–188). Magnesium deficiency is an independent risk factor for migraine (189). Migraine is a common neurological disorder, and its attacks are influenced by nutrients (30).

The United States National Health and Nutrition Study (2021) investigated the relationship between magnesium intake and migraine headaches in 3,626 participants aged 20 to 50 and showed that magnesium intake in both migraine and healthy groups was less than the desired normal amount. According to the results of the study, magnesium deficiency in adults aged 20 to 50, was related to the incidence of migraine (188). In this regard, Assarzadegan et al. (189) compared magnesium serum levels between three groups: (1) healthy persons, (2) people with migraine headaches and during migraine attacks, and (3) those with migraine headaches and between attacks. They concluded that there was a significant difference among the three groups in terms of serum magnesium levels so persons “during attacks” and “between migraine attacks” had lower serum magnesium levels than healthy ones. According to the results of the study, when the magnesium serum level reached below the normal level, the probability of migraine headaches increased 35.3 times (189).

Existing studies have shown the role of magnesium deficiency in migraine pathogenesis through several pathways. Magnesium deficiency in the brain leads to platelet aggregation and the release of glutamate, resulting in the formation of 5-hydroxytryptamine (5-HT). 5-HT acts as a vasoconstrictor at the endpoint and is the factor that facilitates migraine attacks (190, 191). Mitochondrial phosphorylation potential is also decreased in migraine patients due to magnesium deficiency (192).

In their meta-analysis, Chiu et al. (187) compared the effectiveness of intravenous and oral magnesium supplementation in managing migraine patients. The results of the study showed that intravenous magnesium could relieve acute attacks between 15 min and 24 h after infusion, and oral magnesium significantly reduced the frequency and intensity of migraine attacks (187). It seems that the use of magnesium supplements with other drugs, such as sodium valproate and other pain relievers, can be effective in treating migraine. In confirmation of this finding, Khani et al. (193) compared the effectiveness of three types of migraine medications: magnesium and sodium valproate, sodium valproate alone, and magnesium alone. The results of the study demonstrated that magnesium could increase the antimigraine properties of sodium valproate in combined treatment. All migraine characteristics (such as frequency, intensity, duration of attacks and number of medications taken, and migraine disability) were significantly improved in the all groups. But the combination of magnesium and sodium valproate was more effective compared with other groups. Also, magnesium supplements could reduce the dose of valproate to prevent migraine attacks (193).

In general, taking magnesium supplements is effective in treating migraine, and also in combination with antidepressants, and pain relievers can lead to improvement of migraine symptoms. Given that a high percentage of migraine sufferers are deficient in magnesium; therefore, the administration of magnesium supplements can increase the effectiveness of drugs. In this regard, the cellular and molecular studies that investigated the effects of magnesium supplementation on inflammatory and cellular markers were limited. Therefore, we need cellular studies on the mechanism of action to confirm the effectiveness of magnesium supplementation on migraine.

### Probiotics

Probiotics are live microorganisms beneficial to the host when consumed in sufficient amounts. They have been used in treating many sensory and vascular disorders of the brain. Psychobiotics, as a new class of probiotics, are useful for improving brain function and efficiency. They are living bacteria that can have positive, direct, and indirect effects on the function of neurons through cloning in the intestinal flora (194).

Migraine is one of the common vascular diseases of the brain, leading to disturbances in the sensory function of the brain. The pathophysiology of migraine is complex, and the role of genetic, metabolic, and hormonal factors has been proposed (195). Gastrointestinal microbiota, an extensive collection of microorganisms living in the intestine can play an important role in maintaining the normal function of many extra-intestinal organs such as brain. Recently, studies have emphasized the effect of intestinal microbiota on migraine headaches (196). Due to the co-occurrence of migraine and gastrointestinal (GI) disorders, this issue has received more attention. Studies have shown that people who frequently experience digestive disorders have a higher prevalence of headaches (197, 198).

The microbiome-gut-brain content axis consists of three bidirectional, biochemical, signaling pathways between the gut and the brain, and these three pathways include the nervous, endocrine, and immune pathways (198). Studies have suggested mechanisms regarding the interaction between the microbiota and the gut-brain axis. These mechanisms include changes in microbial composition, immune activation, vagus nerve signaling, and the production of specific microbial neuroactive metabolites (197). Intestinal dysbiosis is related to the disruption of hippocampal serotonergic system regulation and the induction of mood and anxiety disorders in animal models (199). The increment in intestinal permeability is another prominent mechanism in the gut-brain axis communication in patients with migraine, leading to inflammation and the release of pro-inflammatory cytokines (200, 201). Cytokines, as important mediators of immune and inflammatory pathways, communicate the function of distant organs, such as the digestive system and the central nervous system (201). High levels of chemokines can lead to the activation of the trigeminal nerve (an essential structure in migraine), the release of vasoactive peptides, and other biochemical mediators like nitric oxide. Eventually, these changes lead to inflammation and migraine attacks (202, 203). Additionally a meta-analysis, (204) showed that the use of probiotic supplements could significantly decrease the levels of CRP and malondialdehyde.

Another factor, connecting the intestine to brain pathways, is severe stress. Stress loads are the most common triggers for migraine attacks. According to the evidence, the hypothalamus-pituitary–adrenal axis changes in migraine patients (205). The stress response includes the release of some hormones like cortisol. Cortisol can lead to an increase in the permeability of the intestinal barrier and allow the transfer of bacteria and pro-inflammatory compounds into the blood (206). One of these compounds is lipopolysaccharide, as a component of gram-negative bacteria with pro-inflammatory effects. Lipopolysaccharides activate pain receptors in trigeminal sensory neurons (207). Recent research has suggested that probiotics can improve intestinal function and amend intestinal leakage caused by stress in animal models and may reduce the level of cortisol in humans (208, 209).

Martami et al. (210) investigated the effectiveness of probiotic capsules/day (14 types of probiotics) on episodic (10 weeks) and chronic migraine patients (8 weeks). They found that the average frequency of migraine attacks, migraine severity, and drug consumption significantly decreased in both patients with episodic and chronic migraine. However, the migraine attack duration decreased only in the chronic migraine patients. Also, there was no significant difference in serum levels of inflammatory markers of migraine patients consuming probiotics (210). In explaining the reason for the different results in the two groups of chronic migraine and episodic migraine, we can pay attention to the results of the study performed by Yong et al. (211). In their study, Yong et al. investigated and compared the intestinal microbiota of people with chronic migraine with those with episodic migraine and healthy people. The results showed that the intestinal microbiota was different in these three groups. The presence of a higher combination of PAC000195_g microbiome was significantly associated with a lower frequency of headaches. The presence of Agatobacter also was significantly associated with the severity of headaches (211). Therefore, receiving psychobiotics with drug interventions can be useful for migraine treatment.

Xie et al. (212) also demonstrated that migraine days, bowel function score, drug consumption, and serum serotonin levels improved in people treated with the combination of elimination diet and probiotics. Ghavami et al. (213) investigated the effectiveness of synbiotic supplementation (12 types of probiotics plus fructooligosaccharides prebiotic, for 12 weeks) on women with migraine. They found that probiotic supplementation significantly reduced the frequency of migraine attacks, the frequency of drug use, digestive problems, zonulin levels, and Hs-CRP levels (213). In contrast, a meta-analysis of RCTs showed that probiotic supplements did not have a significant effect on the frequency and severity of episodic migraine attacks (214).

In this regard, the lack of purity and the same microbial strength in the prescribed supplements has made it difficult to generalize the results of the studies. Thus, the formulation of probiotic products requires checking the purity, strength (amount of living microbes delivered), and composition of the final product (215). The controversy of results may be due to the difference in probiotic supplements in terms of purity, type of species, duration of intervention, and lack of examination of inflammatory serum markers. In general, probiotic supplements have positive effects on migraines; however, larger randomized placebo-controlled trials are warranted.

### Caffeine

Caffeine is a popular and highly consumed food ingredient found in coffee, tea, chocolate, cocoa, some medicines, and dietary supplements (216). Previous studies have shown the beneficial effects of caffeine on several chronic diseases, including cancer, diabetes, cardiovascular disease, and Parkinson’s disease (217). In addition, caffeine may play a role in the treatment of migraine headaches.

Previous evidence has shown that adenosine is one of the neuromodulators involved in the pathophysiology of migraine (218). Since caffeine is structurally similar to adenosine, it competitively antagonizes the effects of adenosine by binding to some of the same receptors. So, it may be effective in the treatment of migraine attacks (219). On the other hand, migraine patients have gastric stasis even outside of acute migraine attacks (220). This decrease in gastric motility in patients with migraine leads to slow absorption of medications and decreases their effectiveness (221). Since caffeine increases gastric motility, it may contribute to its effectiveness when combined with analgesic medicines (222).

In a double-blind randomized placebo-controlled study, a combination of acetaminophen (500 mg), acetylsalicylic acid (500 mg), and caffeine (130 mg) (AAC) was compared with ibuprofen (400 mg) and placebo for acute treatment of migraine. AAC was significantly superior to ibuprofen for pain relief, headache response, and decreasing the functional disability, pain intensity, phonophobia, photophobia, and use of rescue medication (223). Additionally, a meta-analysis illustrated that AAC could be used as the first-line therapy in patients with acute migraine attacks (224). In another randomized clinical trial, Goldstein et al. evaluated the efficacy of a combination product (containing acetaminophen 500 mg, aspirin 500 mg, and caffeine 130 mg) versus sumatriptan (50 mg). Subjects taking combination products had greater headache pain relief scores and lower associated symptoms, use of rescue medication, and disability than those taking sumatriptan (225). Also, another randomized double-blind study compared the efficacy of 100 mg diclofenac sodium soft gel (with or without 100 mg caffeine) with placebo during migraine attacks. Diclofenac soft gel plus caffeine was significantly more effective than a placebo in headache relief (226).

Overall, caffeine (in low doses: 100–130 mg) in combination with analgesic drugs may be effective in treating migraine attacks.

## Limitations

The current narrative review was prepared by carefully screening the related articles and under supervision two expert nutritionist (G.A) and neurologist (F.Kh). However, this is not a systematic review. More studies are required to do a systematic review and meta-analysis to exclusively determine the efficacy, optimum dosages, and duration of supplements. Additionally, the included studies did not report the probable side effects of supplements. Moreover, different risk factors would influence the efficacy of supplements. The efficacy of supplements would be influenced by the interaction between foods, drugs, and other supplements. Moreover, nutrient supplementation would be more effective in patients with nutrient deficiency than other subjects. Because normalization of a deficient nutrient might be as an adjunct therapy. And it might not affect migraine directly. However, the most included studies did not examine the deficiency status of nutrients. Additionally, patients who are exposed to environmental oxidative stress or suffer from genetic deficits (such as methylation pathways) would have more favorites results by related nutrient supplementation, compared with other subjects. Nevertheless, the included studies did not report the potential confounders such as genetic, environmental risk factors, nutrient deficiency or food and drug interactions. Furthermore, some studies investigated the effect of nutrient combinations. Therefore, the effect of each nutrient were not defined.

## Conclusion

Taken together, migraine patients are more prone to have insufficient dietary intakes due to their nausea and vomiting. The deficient or even insufficient levels of vital nutrients would increase the severity and frequency of migraine attacks. Therefore, studies investigated the effect of different nutrients and herbal medicines and indicated that supplementation with riboflavin, omega-3 fatty acids, alpha lipoic acid, magnesium, probiotics, coenzyme Q10, ginger and caffeine would have favorable effects on migraine patients. However, more prospective studies are required to evaluate the effects of other nutrients on migraine patients.

## Glossary

TNF-α: tumor necrosis factor-α
CGRP: calcitonin gene related peptide
COX-2: cyclooxygenase-2
iNOS: inducible nitric oxide synthase
ATP: adenosine tri-phosphate
ROS: reactive oxygen species
FMN: flavin mononucleotide
FAD: flavin– adenosine–dinucleotide
ASA: acetylsalicylic acid
MTHFR: methylenetetrahydrofolate reductase
HDR: headache diary results
DNA: deoxyribonucleic acid
DHF: dihydrofolate
THF: tetrahydrofolic acid
VPA: valproic acid
NO: nitric oxide
OHB12: Hydroxocobalamin
CRPS: complex regional pain syndrome
ROS: reactive oxygen species
nVDR: nuclear vitamin D receptor
MR: Mendelian randomization
IL-6: interleukin 6
MIDAS: migraine disability assessment
PGs: prostaglandins
IL-1: interleukin 1
IFN-8: interferon 8
RBP: Retinol-binding protein
RBP4: retinol-binding protein-4
hs-CRP: high-sensitivity C-reactive protein
NHANES: National Health and Nutrition Survey
RCT: randomized clinical trial
FAs: fatty acids
PUFAs: poly unsaturated fatty acids
EPA: eicosapentaenoic acid
DHA: docosahexaenoic acid
IBD: inflammatory bowel disease
MS: multiple sclerosis
NOS: nitric oxide
LPS: lipopolysaccharides
α-LA: Alpha-lipoic acid
RNS: reactive nitrogen species
MDA: malondialdehyde
CRP: C-reactive protein
DNA: deoxyribonucleic acid
5-HT: 5-hydroxytryptamine
GI: gastrointestinal
CoQ10: Coenzyme Q10
AAC: combination of acetaminophen, acetylsalicylic acid, and caffeine
NF-κB: Nuclear factor-κB
NLRP3: NOD-Like Receptor Protein 3
BFP: body fat percent
HDL-c: high density lipoprotein-cholesterol

## References

[1] Stovner L Hagen K Jensen R Katsarava Z Lipton R Scher A. The global burden of headache: a documentation of headache prevalence and disability worldwide. Cephalalgia. (2007) 27:193–210. doi: 10.1111/j.1468-2982.2007.01288.x, PMID: 17381554

[2] Lewis DW Winner P. The pharmacological treatment options for pediatric migraine: an evidence-based appraisal. NeuroRx. (2006) 3:181–91. doi: 10.1016/j.nurx.2006.01.002, PMID: 16554256 PMC3593442

[3] Pothmann R Danesch U. Migraine prevention in children and adolescents: results of an open study with a special butterbur root extract. Headache. (2005) 45:196–203. doi: 10.1111/j.1526-4610.2005.05044.x 15836592

[4] Larsson B. The role of psychological, health-behaviour and medical factors in adolescent headache. Dev Med Child Neurol. (1988) 30:616–25. doi: 10.1111/j.1469-8749.1988.tb04799.x, PMID: 3229559

[5] Sadeghi O Askari G Maghsoudi Z Nasiri M Khorvash F. Migraine and risk of stroke: review of current evidence. Jundishapur J Chronic Dis Care. (2014) 3:e93799. doi: 10.17795/jjcdc-21707

[6] Moradi F Fazelian S Khorvash F Askari G. The relationship of mood status, quality of life, and dietary intake with migraine symptoms among women with migraine. J Multidiscip Care. (2021) 10:99–104. doi: 10.34172/jmdc.2021.20

[7] Sadeghi O Maghsoudi Z Nasiri M Khorvash F Askari G. The association between anthropometric measurements and severity, frequency and duration of headache attacks in adults with migraine in Isfahan. J Mazandaran Univ Med Sci. (2014) 24:194–203.

[8] Sadeghi O Askari G Maghsoudi Z Ghiasvand R Khorvash F. The association between abdominal obesity and characteristics of migraine attacks in Iranian adults. Iran J Nurs Midwifery Res. (2016) 21:271–7. doi: 10.4103/1735-9066.180378 27186204 PMC4857661

[9] Balali A Karimi E Kazemi M Hadi A Askari G Khorvash F. Associations between diet quality and migraine headaches: a cross-sectional study. Nutr Neurosci. (2024) 27:677–87. doi: 10.1080/1028415X.2023.2244260, PMID: 37542451

[10] Andreou AP Edvinsson L. Mechanisms of migraine as a chronic evolutive condition. J Headache Pain. (2019) 20:117. doi: 10.1186/s10194-019-1066-0 31870279 PMC6929435

[11] Fidan I Yüksel S Ýmir T İrkeç C Aksakal FN. The importance of cytokines, chemokines and nitric oxide in pathophysiology of migraine. J Neuroimmunol. (2006) 171:184–8. doi: 10.1016/j.jneuroim.2005.10.005, PMID: 16325275

[12] Hamed SA. The vascular risk associations with migraine: relation to migraine susceptibility and progression. Atherosclerosis. (2009) 205:15–22. doi: 10.1016/j.atherosclerosis.2008.10.016, PMID: 19054516

[13] Longoni M Ferrarese C. Inflammation and excitotoxicity: role in migraine pathogenesis. Neurol Sci. (2006) 27:s107–10. doi: 10.1007/s10072-006-0582-2, PMID: 16688611

[14] D’Andrea G Leon A. Pathogenesis of migraine: from neurotransmitters to neuromodulators and beyond. Neurol Sci. (2010) 31:1–7. doi: 10.1007/s10072-010-0267-8, PMID: 20464574

[15] Sadeghi O Maghsoudi Z Askari G Khorvash F Feizi A. Association between serum levels of homocysteine with characteristics of migraine attacks in migraine with aura. J Res Med Sci. (2014) 19:1041–5. PMID: 25657748 PMC4310076

[16] Gallagher RM Kunkel R. Migraine medication attributes important for patient compliance: concerns about side effects may delay treatment. Headache. (2003) 43:36–43. doi: 10.1046/j.1526-4610.2003.03006.x 12864756

[17] Whyte CA Tepper SJ. Adverse effects of medications commonly used in the treatment of migraine. Expert Rev Neurother. (2009) 9:1379–91. doi: 10.1586/ern.09.47 19769452

[18] Frank LL. Thiamin in clinical practice. JPEN J Parenter Enteral Nutr. (2015) 39:503–20. doi: 10.1177/0148607114565245 25564426

[19] Hazell AS Butterworth RF. Update of cell damage mechanisms in thiamine deficiency: focus on oxidative stress, excitotoxicity and inflammation. Alcohol Alcohol. (2009) 44:141–7. doi: 10.1093/alcalc/agn120 19151161

[20] Prakash S Kumar Singh A Rathore C. Chronic migraine responding to intravenous thiamine: a report of two cases. Headache. (2016) 56:1204–9. doi: 10.1111/head.12838, PMID: 27197607

[21] Antonio C Massimo T Gianpaolo Z Immacolata PM Erika T. Oral high-dose thiamine improves the symptoms of chronic cluster headache. Case Rep Neurol Med. (2018) 2018:1–5. doi: 10.1155/2018/3901619 PMC5932500 29850313

[22] Montagna P Sacquegna T Martinelli P Cortelli P Bresolin N Moggio M. Mitochondrial abnormalities in migraine. Preliminary findings. Headache. (1988) 28:477–80. doi: 10.1111/j.1526-4610.1988.hed2807477.x 3243709

[23] Stuart S Griffiths L. A possible role for mitochondrial dysfunction in migraine. Mol Gen Genomics. (2012) 287:837–44. doi: 10.1007/s00438-012-0723-7 23052833

[24] Schaefer AM McFarland R Blakely EL He L Whittaker RG Taylor RW. Prevalence of mitochondrial DNA disease in adults. Ann Neurol. (2008) 63:35–9. doi: 10.1002/ana.21217 17886296

[25] Yee AJ. Effectiveness of high-dose riboflavin in migraine prophylaxis. Neurology. (1999) 52:431–2. doi: 10.1212/WNL.52.2.431-a, PMID: 9932987

[26] Kennedy DO. B vitamins and the brain: mechanisms, dose and efficacy—a review. Nutrients. (2016) 8:68. doi: 10.3390/nu8020068, PMID: 26828517 PMC4772032

[27] Nattagh-Eshtivani E Sani MA Dahri M Ghalichi F Ghavami A Arjang P. The role of nutrients in the pathogenesis and treatment of migraine headaches. Biomed Pharmacother. (2018) 102:317–25. doi: 10.1016/j.biopha.2018.03.059, PMID: 29571016

[28] Ramadan NM. Prophylactic migraine therapy: mechanisms and evidence. Curr Pain Headache Rep. (2004) 8:91–5. doi: 10.1007/s11916-004-0022-z 14980143

[29] Sparaco M Feleppa M Lipton R Rapoport A Bigal M. Mitochondrial dysfunction and migraine: evidence and hypotheses. Cephalalgia. (2006) 26:361–72. doi: 10.1111/j.1468-2982.2005.01059.x 16556237

[30] Gross EC Lisicki M Fischer D Sándor PS Schoenen J. The metabolic face of migraine—from pathophysiology to treatment. Nat Rev Neurol. (2019) 15:627–43. doi: 10.1038/s41582-019-0255-4, PMID: 31586135

[31] Powers HJ. Riboflavin (vitamin B-2) and health. Am J Clin Nutr. (2003) 77:1352–60. doi: 10.1093/ajcn/77.6.1352 12791609

[32] Suwannasom N Kao I Pruß A Georgieva R Bäumler H. Riboflavin: the health benefits of a forgotten natural vitamin. Int J Mol Sci. (2020) 21:950. doi: 10.3390/ijms21030950 32023913 PMC7037471

[33] Schoenen J Jacquy J Lenaerts M. Effectiveness of high-dose riboflavin in migraine prophylaxis a randomized controlled trial. Neurology. (1998) 50:466–70. doi: 10.1212/WNL.50.2.466, PMID: 9484373

[34] Boehnke C Reuter U Flach U Schuh-Hofer S Einhäupl K Arnold G. High-dose riboflavin treatment is efficacious in migraine prophylaxis: an open study in a tertiary care Centre. Eur J Neurol. (2004) 11:475–7. doi: 10.1111/j.1468-1331.2004.00813.x, PMID: 15257686

[35] Nambiar N Aiyappa C Srinivasa R. Oral riboflavin versus oral propranolol in migraine prophylaxis: an open label randomized controlled trial. Neurol Asia. (2011) 16:223–229.

[36] Rahimdel A Zeinali A Yazdian-Anari P Hajizadeh R Arefnia E. Effectiveness of vitamin B2 versus sodium valproate in migraine prophylaxis: a randomized clinical trial. Electron Physician. (2015) 7:1344–8. doi: 10.14661/1344 26516440 PMC4623793

[37] Gasperi V Sibilano M Savini I Catani MV. Niacin in the central nervous system: an update of biological aspects and clinical applications. Int J Mol Sci. (2019) 20:974. doi: 10.3390/ijms20040974, PMID: 30813414 PMC6412771

[38] Chong R Wakade C Seamon M Giri B Morgan J Purohit S. Niacin enhancement for Parkinson's disease: an effectiveness trial. Front Aging Neurosci. (2021) 13:667032. doi: 10.3389/fnagi.2021.667032 34220485 PMC8245760

[39] Morris MC Evans DA Bienias JL Scherr PA Tangney CC Hebert LE. Dietary niacin and the risk of incident Alzheimer's disease and of cognitive decline. J Neurol Neurosurg Psychiatry. (2004) 75:1093–9. doi: 10.1136/jnnp.2003.025858, PMID: 15258207 PMC1739176

[40] Rawji KS Young AMH Ghosh T Michaels NJ Mirzaei R Kappen J. Niacin-mediated rejuvenation of macrophage/microglia enhances remyelination of the aging central nervous system. Acta Neuropathol. (2020) 139:893–909. doi: 10.1007/s00401-020-02129-7, PMID: 32030468 PMC7181452

[41] Velling DA Dodick DW Muir JJ. Sustained-release niacin for prevention of migraine headache. Mayo Clin Proc. (2003) 78:770–1. doi: 10.4065/78.6.770 12934790

[42] Gedye A. Hypothesized treatment for migraines using low doses of tryptophan, niacin, calcium, caffeine, and acetylsalicylic acid. Med Hypotheses. (2001) 56:91–4. doi: 10.1054/mehy.2000.1117, PMID: 11133261

[43] Tepper SJ Rapoport A Sheftell F. The pathophysiology of migraine. Neurologist. (2001) 7:279–86. doi: 10.1097/00127893-200109000-00002 12803669

[44] Marriage B Clandinin MT Glerum DM. Nutritional cofactor treatment in mitochondrial disorders. J Am Diet Assoc. (2003) 103:1029–38. doi: 10.1016/S0002-8223(03)00476-0 12891154

[45] Sanvictores T Chauhan S. Vitamin B5 (pantothenic acid). In: StatPearls. Treasure Island (FL): StatPearls publishing, copyright © 2023, StatPearls Publishing LLC. (2023) 33085380

[46] Gheita AA Gheita TA Kenawy SA. The potential role of B5: a stitch in time and switch in cytokine. Phytother Res. (2020) 34:306–14. doi: 10.1002/ptr.6537, PMID: 31691401

[47] Hessen E Lossius MI Reinvang I Gjerstad L. Predictors of neuropsychological impairment in seizure-free epilepsy patients. Epilepsia. (2006) 47:1870–8. doi: 10.1111/j.1528-1167.2006.00830.x, PMID: 17116027

[48] Xu J Patassini S Begley P Church S Waldvogel HJ Faull RLM. Cerebral deficiency of vitamin B5 (d-pantothenic acid; pantothenate) as a potentially-reversible cause of neurodegeneration and dementia in sporadic Alzheimer's disease. Biochem Biophys Res Commun. (2020) 527:676–81. doi: 10.1016/j.bbrc.2020.05.015 32416962

[49] Patassini S Begley P Xu J Church SJ Kureishy N Reid SJ. Cerebral vitamin B5 (D-pantothenic acid) deficiency as a potential cause of metabolic perturbation and neurodegeneration in Huntington's disease. Meta. (2019) 9:113. doi: 10.3390/metabo9060113, PMID: 31212603 PMC6630497

[50] Bais S Rana RK Dongre N Goyanar G Panwar AS Soni AK. Protective effect of pantothenic acid in kainic acid-induced status eilepticus and associated neurodegeneration in mice. Adv Neuro. (2022) 1:40. doi: 10.36922/an.v1i2.40

[51] Gaby AR. Intravenous nutrient therapy: the "Myers' cocktail". Altern Med Rev. (2002) 7:389–403. 12410623

[52] Shtyrlin YG Petukhov A Strelnik A Shtyrlin N Iksanova A Pugachev M. Chemistry of pyridoxine in drug design. Russ Chem Bull. (2019) 68:911–45. doi: 10.1007/s11172-019-2504-5

[53] Hartvig P Lindner K Bjurling P Långström B Tedroff J. Pyridoxine effect on synthesis rate of serotonin in the monkey brain measured with positron emission tomography. J Neural Transm Gen Sect. (1995) 102:91–7. doi: 10.1007/BF01276505, PMID: 8748674

[54] Lea R Colson N Quinlan S Macmillan J Griffiths L. The effects of vitamin supplementation and MTHFR (C677T) genotype on homocysteine-lowering and migraine disability. Pharmacogenet Genomics. (2009) 19:422–8. doi: 10.1097/FPC.0b013e32832af5a3, PMID: 19384265

[55] Christen WG Glynn RJ Chew EY Albert CM Manson JE. Folic acid, pyridoxine, and cyanocobalamin combination treatment and age-related macular degeneration in women: the Women's antioxidant and folic acid cardiovascular study. Arch Intern Med. (2009) 169:335–41. doi: 10.1001/archinternmed.2008.574, PMID: 19237716 PMC2648137

[56] Rubino E Ferrero M Rainero I Binello E Vaula G Pinessi L. Association of the C677T polymorphism in the MTHFR gene with migraine: a meta-analysis. Cephalalgia. (2009) 29:818–25. doi: 10.1111/j.1468-2982.2007.01400.x 17714520

[57] Frosst P Blom H Milos R Goyette P Sheppard CA Matthews R. A candidate genetic risk factor for vascular disease: a common mutation in methylenetetrahydrofolate reductase. Nat Genet. (1995) 10:111–3. doi: 10.1038/ng0595-111, PMID: 7647779

[58] Miya Shaik M Lin Tan HA Kamal M Hua Gan S. Do folate, vitamins B6 and B12 play a role in the pathogenesis of migraine? The role of pharmacoepigenomics. CNS Neurol Disord Drug Targets. (2014) 13:828–35. doi: 10.2174/18715273113129990112 24040787

[59] Sadeghi O Nasiri M Maghsoudi Z Pahlavani N Rezaie M Askari G. Effects of pyridoxine supplementation on severity, frequency and duration of migraine attacks in migraine patients with aura: a double-blind randomized clinical trial study in Iran. Iran J Neurol. (2015) 14:74–80. PMID: 26056551 PMC4449397

[60] Menon S Lea RA Roy B Hanna M Wee S Haupt LM. Genotypes of the MTHFR C677T and MTRR A66G genes act independently to reduce migraine disability in response to vitamin supplementation. Pharmacogenet Genomics. (2012) 22:741–9. doi: 10.1097/FPC.0b013e3283576b6b, PMID: 22926161

[61] Said HM. Biotin: biochemical, physiological and clinical aspects. Subcell Biochem. (2012) 56:1–19. doi: 10.1007/978-94-007-2199-9_1, PMID: 22116691

[62] Saleem F Soos MP. Biotin Deficiency. In: StatPearls. Treasure Island (FL): StatPearls Publishing, Copyright © 2023, StatPearls Publishing LLC. (2023)

[63] Anagnostouli M Livaniou E Nyalala JO Evangelatos G Zournas C Ithakissios DS. Cerebrospinal fluid levels of biotin in various neurological disorders. Acta Neurol Scand. (1999) 99:387–92. doi: 10.1111/j.1600-0404.1999.tb07369.x 10577274

[64] Tourbah A Lebrun-Frenay C Edan G Clanet M Papeix C Vukusic S. MD1003 (high-dose biotin) for the treatment of progressive multiple sclerosis: a randomised, double-blind, placebo-controlled study. Mult Scler. (2016) 22:1719–31. doi: 10.1177/1352458516667568, PMID: 27589059 PMC5098693

[65] Scaglione F Panzavolta G. Folate, folic acid and 5-methyltetrahydrofolate are not the same thing. Xenobiotica. (2014) 44:480–8. doi: 10.3109/00498254.2013.845705 24494987

[66] Mitchell LE Adzick NS Melchionne J Pasquariello PS Sutton LN Whitehead AS. Spina bifida. Lancet. (2004) 364:1885–95. doi: 10.1016/S0140-6736(04)17445-X 15555669

[67] Reisch HS Flynn MA. Folic acid and the prevention of neural tube defects (NTDs) challenges and recommendations for public health. Can J Public Health. (2002) 93:254–8. doi: 10.1007/BF03405011, PMID: 12154525 PMC6980045

[68] Sadeghi O Maghsoudi Z Khorvash F Ghiasvand R Askari G. Assessment of pyridoxine and folate intake in migraine patients. Adv Biomed Res. (2016) 5:47. doi: 10.4103/2277-9175.178800 27110544 PMC4817396

[69] Fila M Pawłowska E Blasiak J. Mitochondria in migraine pathophysiology–does epigenetics play a role? Arch Med Sci. (2019) 15:944–56. doi: 10.5114/aoms.2019.86061, PMID: 31360189 PMC6657237

[70] Fila M Chojnacki C Chojnacki J Blasiak J. Is an “epigenetic diet” for migraines justified? The case of folate and DNA methylation. Nutrients. (2019) 11:2763. doi: 10.3390/nu11112763, PMID: 31739474 PMC6893742

[71] Fila M Chojnacki C Chojnacki J Blasiak J. Nutrients to improve mitochondrial function to reduce brain energy deficit and oxidative stress in migraine. Nutrients. (2021) 13:4433. doi: 10.3390/nu13124433, PMID: 34959985 PMC8707228

[72] Askari G Nasiri M Mozaffari-Khosravi H Rezaie M Bagheri-Bidakhavidi M Sadeghi O. The effects of folic acid and pyridoxine supplementation on characteristics of migraine attacks in migraine patients with aura: a double-blind, randomized placebo-controlled, clinical trial. Nutrition. (2017) 38:74–9. doi: 10.1016/j.nut.2017.01.007, PMID: 28526386

[73] Nematgorgani S Razeghi-Jahromi S Jafari E Togha M Rafiee P Ghorbani Z. B vitamins and their combination could reduce migraine headaches: a randomized double-blind controlled trial. Curr J Neurol. (2022) 21:105–18. doi: 10.18502/cjn.v21i2.10494, PMID: 38011468 PMC9860208

[74] Menon S Nasir B Avgan N Ghassabian S Oliver C Lea R. The effect of 1 mg folic acid supplementation on clinical outcomes in female migraine with aura patients. J Headache Pain. (2016) 17:1–7. doi: 10.1186/s10194-016-0652-7 PMC4919187 27339806

[75] Stabler SP. Vitamin B12 deficiency. N Engl J Med. (2013) 368:149–60. doi: 10.1056/NEJMcp1113996 23301732

[76] Green R. Vitamin B12 deficiency from the perspective of a practicing hematologist. Blood. (2017) 129:2603–11. doi: 10.1182/blood-2016-10-569186 28360040

[77] Olesen J Thomsen L Lassen L Olesen I. The nitric oxide hypothesis of migraine and other vascular headaches. Cephalalgia. (1995) 15:94–100. doi: 10.1046/j.1468-2982.1995.015002094.x, PMID: 7641257

[78] Rajanayagam M Li C Rand M. Differential effects of hydroxocobalamin on NO-mediated relaxations in rat aorta and anococcygeus muscle. Br J Pharmacol. (1993) 108:3–5. doi: 10.1111/j.1476-5381.1993.tb13429.x, PMID: 8428210 PMC1907706

[79] Van der Kuy P Merkus F Russel F Lohman J Hooymans P. Bioavailability of oral hydroxocobalamin. Br J Clin Pharmacol. (2000) 49:395P–6P.

[80] Slot WB Merkus F Van Deventer S Tytgat G. Normalization of plasma vitamin B12 concentration by intranasal hydroxocobalamin in vitamin B12-deficient patients. Gastroenterology. (1997) 113:430–3. doi: 10.1053/gast.1997.v113.pm9247460, PMID: 9247460

[81] Van Asselt D Merkus F Russel F Hoefnagels W. Nasal absorption of hydroxocobalamin in healthy elderly adults. Br J Clin Pharmacol. (1998) 45:83–6. doi: 10.1046/j.1365-2125.1998.00642.x, PMID: 9489599 PMC1873990

[82] Van der Kuy P Merkus F Russel F Lohman J Hooymans P. Pharmacokinetics of intranasal and oral hydroxocobalamin in healthy subjects. Br J Clin Pharmacol. (2001) 51:505P.

[83] Van der Kuy P-H Merkus F Lohman J Jt B Hooymans P. Hydroxocobalamin, a nitric oxide scavenger, in the prophylaxis of migraine: an open, pilot study. Cephalalgia. (2002) 22:513–9. doi: 10.1046/j.1468-2982.2002.00412.x, PMID: 12230592

[84] Grosso G Bei R Mistretta A Marventano S Calabrese G Masuelli L. Effects of vitamin C on health: a review of evidence. Front Biosci (Landmark Ed). (2013) 18:1017–29. doi: 10.2741/4160, PMID: 23747864

[85] Kocot J Luchowska-Kocot D Kiełczykowska M Musik I Kurzepa J. Does vitamin C influence neurodegenerative diseases and psychiatric disorders? Nutrients. (2017) 9:659. doi: 10.3390/nu9070659, PMID: 28654017 PMC5537779

[86] Eldridge CF Bunge MB Bunge RP Wood PM. Differentiation of axon-related Schwann cells in vitro. I. Ascorbic acid regulates basal lamina assembly and myelin formation. J Cell Biol. (1987) 105:1023–34. doi: 10.1083/jcb.105.2.1023, PMID: 3624305 PMC2114758

[87] Besler HT Comoğlu S Okçu Z. Serum levels of antioxidant vitamins and lipid peroxidation in multiple sclerosis. Nutr Neurosci. (2002) 5:215–20. doi: 10.1080/10284150290029205, PMID: 12041878

[88] Polachini CR Spanevello RM Zanini D Baldissarelli J Pereira LB Schetinger MR. Evaluation of Delta-Aminolevulinic dehydratase activity, oxidative stress biomarkers, and vitamin D levels in patients with multiple sclerosis. Neurotox Res. (2016) 29:230–42. doi: 10.1007/s12640-015-9584-2, PMID: 26690779

[89] Al-Himyari F. Supplementary vitamin C in the treatment of Parkinson's disease. J Kerbala Univ. (2009) 5:186–8.

[90] Visser EJ Drummond PD Lee-Visser JLA. Reduction in migraine and headache frequency and intensity with combined antioxidant prophylaxis (N-acetylcysteine, vitamin E, and vitamin C): a randomized sham-controlled pilot study. Pain Pract. (2020) 20:737–47. doi: 10.1111/papr.12902 32306462

[91] Chayasirisobhon S. Use of a pine bark extract and antioxidant vitamin combination product as therapy for migraine in patients refractory to pharmacologic medication. Headache. (2006) 46:788–93. doi: 10.1111/j.1526-4610.2006.00454.x 16643582

[92] Chayasirisobhon S. Efficacy of *Pinus radiata* bark extract and vitamin C combination product as a prophylactic therapy for recalcitrant migraine and long-term results. Acta Neurol Taiwanica. (2013) 22:13–21. PMID: 23479241

[93] de Mos M Huygen F Dieleman JP Koopman J Stricker CBH Sturkenboom M. Medical history and the onset of complex regional pain syndrome (CRPS). Pain. (2008) 139:458–66. doi: 10.1016/j.pain.2008.07.002, PMID: 18760877

[94] Padayatty SJ Katz A Wang Y Eck P Kwon O Lee JH. Vitamin C as an antioxidant: evaluation of its role in disease prevention. J Am Coll Nutr. (2003) 22:18–35. doi: 10.1080/07315724.2003.10719272 12569111

[95] Youness RA Dawoud A ElTahtawy O Farag MA. Fat-soluble vitamins: updated review of their role and orchestration in human nutrition throughout life cycle with sex differences. Nutr Metab. (2022) 19:60. doi: 10.1186/s12986-022-00696-y, PMID: 36064551 PMC9446875

[96] Maresz K. Growing evidence of a proven mechanism shows vitamin K2 can impact health conditions beyond bone and cardiovascular. Integr Med (Encinitas). (2021) 20:34–8. PMID: 34602875 PMC8483258

[97] Sconce E Avery P Wynne H Kamali F. Vitamin K supplementation can improve stability of anticoagulation for patients with unexplained variability in response to warfarin. Blood. (2007) 109:2419–23. doi: 10.1182/blood-2006-09-049262, PMID: 17110451

[98] Mansour AG Ahdab R Daaboul Y Korjian S Morrison DA Hariri E. Vitamin K2 status and arterial stiffness among untreated migraine patients: a case-control study. Headache. (2020) 60:589–99. doi: 10.1111/head.13715 31769041

[99] Hadipour E Tayarani-Najaran Z Fereidoni M. Vitamin K2 protects PC12 cells against Aβ (1-42) and H2O2-induced apoptosis via p38 MAP kinase pathway. Nutr Neurosci. (2020) 23:343–52. doi: 10.1080/1028415X.2018.1504428, PMID: 30058479

[100] Halder M Petsophonsakul P Akbulut AC Pavlic A Bohan F Anderson E. Vitamin K: double bonds beyond coagulation insights into differences between vitamin K1 and K2 in health and disease. Int J Mol Sci. (2019) 20:896. doi: 10.3390/ijms20040896, PMID: 30791399 PMC6413124

[101] Holick MF. The vitamin D deficiency pandemic: approaches for diagnosis, treatment and prevention. Rev Endocr Metab Disord. (2017) 18:153–65. doi: 10.1007/s11154-017-9424-1, PMID: 28516265

[102] Ao T Kikuta J Ishii M. The effects of vitamin D on immune system and inflammatory diseases. Biomol Ther. (2021) 11:1624. doi: 10.3390/biom11111624, PMID: 34827621 PMC8615708

[103] Colotta F Jansson B Bonelli F. Modulation of inflammatory and immune responses by vitamin D. J Autoimmun. (2017) 85:78–97. doi: 10.1016/j.jaut.2017.07.007 28733125

[104] Rapisarda L Mazza MR Tosto F Gambardella A Bono F Sarica A. Relationship between severity of migraine and vitamin D deficiency: a case-control study. Neurol Sci. (2018) 39:167–8. doi: 10.1007/s10072-018-3384-4, PMID: 29904877

[105] Mottaghi T Askari G Khorvash F Maracy MR. Effect of vitamin D supplementation on symptoms and C-reactive protein in migraine patients. J Res Med Sci. (2015) 20:477–82. doi: 10.4103/1735-1995.163971, PMID: 26487877 PMC4590203

[106] Niu P-P Wang X Xu Y-M. Higher circulating vitamin D levels are associated with decreased migraine risk: a Mendelian randomization study. Front Nutr. (2022) 9:907789. doi: 10.3389/fnut.2022.907789, PMID: 36159496 PMC9505695

[107] Susanti R Syafrita Y Rita RS Darwin E Lipoeto NI Ali H. The influence of vitamin D3 administration on the levels of CGRP, glutamate, and NLRP3 during the ictal phase in chronic migraine patients. Pharm J. (2023) 15:1052–8. doi: 10.5530/pj.2023.15.193

[108] Ghorbani Z Togha M Rafiee P Ahmadi ZS Rasekh Magham R Djalali M. Vitamin D3 might improve headache characteristics and protect against inflammation in migraine: a randomized clinical trial. Neurol Sci. (2020) 41:1183–92. doi: 10.1007/s10072-019-04220-8, PMID: 31897949

[109] Hu C Fan Y Wu S Zou Y Qu X. Vitamin D supplementation for the treatment of migraine: a meta-analysis of randomized controlled studies. Am J Emerg Med. (2021) 50:784–8. doi: 10.1016/j.ajem.2021.07.062, PMID: 34879503

[110] Ghorbani Z Togha M Rafiee P Ahmadi ZS Rasekh Magham R Haghighi S. Vitamin D in migraine headache: a comprehensive review on literature. Neurol Sci. (2019) 40:2459–77. doi: 10.1007/s10072-019-04021-z, PMID: 31377873

[111] Wu D Liu L Meydani M Meydani SN. Vitamin E increases production of vasodilator prostanoids in human aortic endothelial cells through opposing effects on cyclooxygenase-2 and phospholipase A2. J Nutr. (2005) 135:1847–53. doi: 10.1093/jn/135.8.1847, PMID: 16046707

[112] Armagan HH Karaman K Yilmaz DY. Antioxidant and cytokine levels in plasma of patients with attack and non-attack periods. J Cell Neurosci Oxid Stress. (2020) 12:914–21. doi: 10.37212/jcnos.806797

[113] Nazıroğlu M Çelik Ö Uğuz AC Bütün A. Protective effects of riboflavin and selenium on brain microsomal ca 2+-ATPase and oxidative damage caused by glyceryl trinitrate in a rat headache model. Biol Trace Elem Res. (2015) 164:72–9. doi: 10.1007/s12011-014-0199-x, PMID: 25492827

[114] Ziaei S Kazemnejad A Sedighi A. The effect of vitamin E on the treatment of menstrual migraine. Med Sci Monit. (2009) 15:CR16-9. 19114966

[115] Cupini LM Corbelli I Sarchelli P. Menstrual migraine: what it is and does it matter? J Neurol. (2021) 268:2355–63. doi: 10.1007/s00415-020-09726-2, PMID: 31989282

[116] Oliveira LM Teixeira FME Sato MN. Impact of retinoic acid on immune cells and inflammatory diseases. Mediat Inflamm. (2018) 2018:1–17. doi: 10.1155/2018/3067126 PMC6109577 30158832

[117] Erkelens MN Mebius RE. Retinoic acid and immune homeostasis: a balancing act. Trends Immunol. (2017) 38:168–80. doi: 10.1016/j.it.2016.12.006, PMID: 28094101

[118] Pang X-Y Wang S Jurczak MJ Shulman GI Moise AR. Retinol saturase modulates lipid metabolism and the production of reactive oxygen species. Arch Biochem Biophys. (2017) 633:93–102. doi: 10.1016/j.abb.2017.09.009 28927883 PMC5659944

[119] Zhong M Kawaguchi R Costabile B Tang Y Hu J Cheng G. Regulatory mechanism for the transmembrane receptor that mediates bidirectional vitamin a transport. Proc Natl Acad Sci. (2020) 117:9857–64. doi: 10.1073/pnas.1918540117, PMID: 32300017 PMC7211970

[120] Tanik N Celikbilek A Metin A Gocmen AY Inan LE. Retinol-binding protein-4 and hs-CRP levels in patients with migraine. Neurol Sci. (2015) 36:1823–7. doi: 10.1007/s10072-015-2262-6, PMID: 26012852

[121] Togha M Razeghi Jahromi S Ghorbani Z Ghaemi A Rafiee P. An investigation of oxidant/antioxidant balance in patients with migraine: a case-control study. BMC Neurol. (2019) 19:1–10. doi: 10.1186/s12883-019-1555-4 31837702 PMC6911287

[122] Tripathi GM Kalita J Misra UK. A study of oxidative stress in migraine with special reference to prophylactic therapy. Int J Neurosci. (2018) 128:318–24. doi: 10.1080/00207454.2017.1374959, PMID: 28903615

[123] Peng C Gao L Wu K Jiang X Chen X Li C. Association between the prognostic nutritional index and severe headache or migraine: a population-based study. Nutr Neurosci. (2022) 26:1202–11. doi: 10.1080/1028415X.2022.2143958 36384436

[124] Alemany R Perona JS Sánchez-Dominguez JM Montero E Cañizares J Bressani R. G protein-coupled receptor systems and their lipid environment in health disorders during aging. Biochim Biophys Acta. (2007) 1768:964–75. doi: 10.1016/j.bbamem.2006.09.024, PMID: 17070497

[125] Bazan NG. Omega-3 fatty acids, pro-inflammatory signaling and neuroprotection. Curr Opin Clin Nutr Metab Care. (2007) 10:136–41. doi: 10.1097/MCO.0b013e32802b7030 17285000

[126] Forman MS Lal D Zhang B Dabir DV Swanson E Lee VM-Y. Transgenic mouse model of tau pathology in astrocytes leading to nervous system degeneration. J Neurosci. (2005) 25:3539–50. doi: 10.1523/JNEUROSCI.0081-05.2005 15814784 PMC6725385

[127] Simopoulos AP. Omega-3 fatty acids in inflammation and autoimmune diseases. J Am Coll Nutr. (2002) 21:495–505. doi: 10.1080/07315724.2002.10719248, PMID: 12480795

[128] Wall R Ross RP Fitzgerald GF Stanton C. Fatty acids from fish: the anti-inflammatory potential of long-chain omega-3 fatty acids. Nutr Rev. (2010) 68:280–9. doi: 10.1111/j.1753-4887.2010.00287.x 20500789

[129] Calder PC. N− 3 polyunsaturated fatty acids, inflammation, and inflammatory diseases. Am J Clin Nutr. (2006) 83:1505S–19S. doi: 10.1093/ajcn/83.6.1505S 16841861

[130] Xu Z-Z Zhang L Liu T Park JY Berta T Yang R. Resolvins RvE1 and RvD1 attenuate inflammatory pain via central and peripheral actions. Nat Med. (2010) 16:592–7. doi: 10.1038/nm.2123, PMID: 20383154 PMC2866054

[131] Corsi L Momo Dongmo B Avallone R. Supplementation of omega 3 fatty acids improves oxidative stress in activated BV2 microglial cell line. Int J Food Sci Nutr. (2015) 66:293–9. doi: 10.3109/09637486.2014.986073 25582176

[132] Layé S. Polyunsaturated fatty acids, neuroinflammation and well being. Prostaglandins Leukot Essent Fatty Acids. (2010) 82:295–303. doi: 10.1016/j.plefa.2010.02.006 20227866

[133] Pradalier A Bakouche P Baudesson G Delage A Cornaille-Lafage G Launay J. Failure of omega-3 polyunsaturated fatty acids in prevention of migraine: a double-blind study versus placebo. Cephalalgia. (2001) 21:818–22. doi: 10.1046/j.1468-2982.2001.218240.x 11737007

[134] Tajmirriahi M Sohelipour M Basiri K Shaygannejad V Ghorbani A Saadatnia M. The effects of sodium valproate with fish oil supplementation or alone in migraine prevention: a randomized single-blind clinical trial. Iran J Neurol. (2012) 11:21–4. PMID: 24250854 PMC3829229

[135] Wang H-F Liu W-C Zailani H Yang C-C Chen T-B Chang C-M. A 12-week randomized double-blind clinical trial of eicosapentaenoic acid intervention in episodic migraine. Brain Behav Immun. (2024) 118:459–67. doi: 10.1016/j.bbi.2024.03.019, PMID: 38499208

[136] Ramsden CE Faurot KR Zamora D Suchindran CM MacIntosh BA Gaylord S. Targeted alteration of dietary n-3 and n-6 fatty acids for the treatment of chronic headaches: a randomized trial. Pain. (2013) 154:2441–51. doi: 10.1016/j.pain.2013.07.028, PMID: 23886520 PMC3850757

[137] Maghsoumi-Norouzabad L Mansoori A Abed R Shishehbor F. Effects of omega-3 fatty acids on the frequency, severity, and duration of migraine attacks: a systematic review and meta-analysis of randomized controlled trials. Nutr Neurosci. (2018) 21:614–23. doi: 10.1080/1028415X.2017.1344371, PMID: 28665211

[138] Djalali M Talebi S Djalali E Abdolahi M Travica N Djalali M. The effect of omega-3 fatty acids supplementation on inflammatory biomarkers in subjects with migraine: a randomized, double-blind, placebo-controlled trial. Immunopharmacol Immunotoxicol. (2023) 45:565–70. doi: 10.1080/08923973.2023.2196600 37126739

[139] Salehi B Berkay Yılmaz Y Antika G Boyunegmez Tumer T Fawzi Mahomoodally M Lobine D. Insights on the use of α-lipoic acid for therapeutic purposes. Biomol Ther. (2019) 9:356. doi: 10.3390/biom9080356, PMID: 31405030 PMC6723188

[140] Jalilpiran Y Hajishafiee M Khorshidi M Rezvani H Mohammadi-Sartang M Rahmani J. The effect of alpha-lipoic acid supplementation on endothelial function: a systematic review and meta-analysis. Phytother Res. (2021) 35:2386–95. doi: 10.1002/ptr.6959, PMID: 33205568

[141] Borkum JM. Brain energy deficit as a source of oxidative stress in migraine: a molecular basis for migraine susceptibility. Neurochem Res. (2021) 46:1913–32. doi: 10.1007/s11064-021-03335-9, PMID: 33939061

[142] Borkum JM. The migraine attack as a homeostatic, neuroprotective response to brain oxidative stress: preliminary evidence for a theory. Headache. (2018) 58:118–35. doi: 10.1111/head.13214 29034461

[143] Vollono C Primiano G Della Marca G Losurdo A Servidei S. Migraine in mitochondrial disorders: prevalence and characteristics. Cephalalgia. (2018) 38:1093–106. doi: 10.1177/0333102417723568 28762753

[144] Younis S Hougaard A Vestergaard MB Larsson HB Ashina M. Migraine and magnetic resonance spectroscopy: a systematic review. Curr Opin Neurol. (2017) 30:246–62. doi: 10.1097/WCO.0000000000000436, PMID: 28240609

[145] Zonooz SR Hasani M Morvaridzadeh M Pizarro AB Heydari H Yosaee S. Effect of alpha-lipoic acid on oxidative stress parameters: a systematic review and meta-analysis. J Funct Foods. (2021) 87:104774. doi: 10.1016/j.jff.2021.104774

[146] Eren Y Dirik E Neşelioğlu S Erel Ö. Oxidative stress and decreased thiol level in patients with migraine: cross-sectional study. Acta Neurol Belg. (2015) 115:643–9. doi: 10.1007/s13760-015-0427-y, PMID: 25595415

[147] Gross EC Putananickal N Orsini A-L Vogt DR Sandor PS Schoenen J. Mitochondrial function and oxidative stress markers in higher-frequency episodic migraine. Sci Rep. (2021) 11:4543. doi: 10.1038/s41598-021-84102-2, PMID: 33633187 PMC7907128

[148] Rezaei Kelishadi M Alavi Naeini A Askari G Khorvash F Heidari Z. The efficacy of alpha-lipoic acid in improving oxidative, inflammatory, and mood status in women with episodic migraine in a randomised, double-blind, placebo-controlled clinical trial. Int J Clin Pract. (2021) 75:e14455. doi: 10.1111/ijcp.14455 34105866

[149] Cavestro C Bedogni G Molinari F Mandrino S Rota E Frigeri MC. Alpha-lipoic acid shows promise to improve migraine in patients with insulin resistance: a 6-month exploratory study. J Med Food. (2018) 21:269–73. doi: 10.1089/jmf.2017.0068, PMID: 28976801

[150] Magis D Ambrosini A Sándor P Jacquy J Laloux P Schoenen J. A randomized double-blind placebo-controlled trial of thioctic acid in migraine prophylaxis. Headache. (2007) 47:52–7. doi: 10.1111/j.1526-4610.2006.00626.x 17355494

[151] Fogacci F Rizzo M Krogager C Kennedy C Georges CM Knežević T. Safety evaluation of α-lipoic acid supplementation: a systematic review and meta-analysis of randomized placebo-controlled clinical studies. Antioxidants. (2020) 9:1011. doi: 10.3390/antiox9101011 33086555 PMC7603186

[152] Raizner AE. Coenzyme Q(10). Methodist Debakey Cardiovasc J. (2019) 15:185–91. doi: 10.14797/mdcj-15-3-185, PMID: 31687097 PMC6822644

[153] Garrido-Maraver J Cordero MD Oropesa-Ávila M Fernández Vega A de la Mata M Delgado Pavón A. Coenzyme q10 therapy. Mol Syndromol. (2014) 5:187–97. doi: 10.1159/000360101, PMID: 25126052 PMC4112525

[154] Garrido-Maraver J Cordero MD Oropesa-Avila M Vega AF de la Mata M Pavon AD. Clinical applications of coenzyme Q10. Front Biosci (Landmark Ed). (2014) 19:619–33. doi: 10.2741/4231 24389208

[155] Yorns WR Jr Hardison HH. Mitochondrial dysfunction in migraine. Semin Pediatr Neurol. (2013) 20:188–93. doi: 10.1016/j.spen.2013.09.002 24331360

[156] Bohra SK Achar RR Chidambaram SB Pellegrino C Laurin J Masoodi M. Current perspectives on mitochondrial dysfunction in migraine. Eur J Neurosci. (2022) 56:3738–54. doi: 10.1111/ejn.15676 35478208

[157] Pradhan N Singh C Singh A. Coenzyme Q10 a mitochondrial restorer for various brain disorders. Naunyn Schmiedeberg's Arch Pharmacol. (2021) 394:2197–222. doi: 10.1007/s00210-021-02161-8, PMID: 34596729

[158] Moskowitz MA. Neurogenic inflammation in the pathophysiology and treatment of migraine. Neurology. (1993) 43:S16–20. PMID: 8389008

[159] Tietjen GE Khubchandani J. Vascular biomarkers in migraine. Cephalalgia. (2015) 35:95–117. doi: 10.1177/0333102414544976 25281220

[160] Edvinsson L. The Trigeminovascular pathway: role of CGRP and CGRP receptors in migraine. Headache. (2017) 57:47–55. doi: 10.1111/head.13081 28485848

[161] Karsan N Goadsby PJ. CGRP mechanism antagonists and migraine management. Curr Neurol Neurosci Rep. (2015) 15:25. doi: 10.1007/s11910-015-0547-z 25790955

[162] Donnino MW Cocchi MN Salciccioli JD Kim D Naini AB Buettner C. Coenzyme Q10 levels are low and may be associated with the inflammatory cascade in septic shock. Crit Care. (2011) 15:R189. doi: 10.1186/cc10343, PMID: 21827677 PMC3271709

[163] Dahri M Tarighat-Esfanjani A Asghari-Jafarabadi M Hashemilar M. Oral coenzyme Q10 supplementation in patients with migraine: effects on clinical features and inflammatory markers. Nutr Neurosci. (2019) 22:607–15. doi: 10.1080/1028415X.2017.1421039 29298622

[164] Shoeibi A Olfati N Soltani Sabi M Salehi M Mali S Akbari OM. Effectiveness of coenzyme Q10 in prophylactic treatment of migraine headache: an open-label, add-on, controlled trial. Acta Neurol Belg. (2017) 117:103–9. doi: 10.1007/s13760-016-0697-z, PMID: 27670440

[165] Dahri M Sadeghi AS Pahlavani N Nattagh-Eshtivani E Hashemilar M Asghari-Jafarabadi M. The effects of coenzyme Q10 supplementation on oxidative status and lipid profile in migraine patients: a randomized double-blinded controlled clinical trial. Clin Nutr Res. (2023) 12:257–68. doi: 10.7762/cnr.2023.12.4.257, PMID: 37969937 PMC10641325

[166] Hajihashemi P Askari G Khorvash F Reza Maracy M Nourian M. The effects of concurrent coenzyme Q10, L-carnitine supplementation in migraine prophylaxis: a randomized, placebo-controlled, double-blind trial. Cephalalgia. (2019) 39:648–54. doi: 10.1177/0333102418821661 30612463

[167] Sazali S Badrin S Norhayati MN Idris NS. Coenzyme Q10 supplementation for prophylaxis in adult patients with migraine-a meta-analysis. BMJ Open. (2021) 11:e039358. doi: 10.1136/bmjopen-2020-039358, PMID: 33402403 PMC7786797

[168] Parohan M Sarraf P Javanbakht MH Ranji-Burachaloo S Djalali M. Effect of coenzyme Q10 supplementation on clinical features of migraine: a systematic review and dose-response meta-analysis of randomized controlled trials. Nutr Neurosci. (2020) 23:868–75. doi: 10.1080/1028415X.2019.1572940, PMID: 30727862

[169] Taha M Abdulwahhab M Mostafa A. The effect of coenzyme Q10 as a prophylactic treatment in episodic migraine. Duzce Med J. (2023) 25:147–51. doi: 10.18678/dtfd.1229687

[170] Kiyama R. Nutritional implications of ginger: chemistry, biological activities and signaling pathways. J Nutr Biochem. (2020) 86:108486. doi: 10.1016/j.jnutbio.2020.108486 32827666

[171] Ballester P Cerdá B Arcusa R Marhuenda J Yamedjeu K Zafrilla P. Effect of ginger on inflammatory diseases. Molecules. (2022) 27:27(21). doi: 10.3390/molecules27217223 PMC9654013 36364048

[172] Roudsari NM Lashgari NA Momtaz S Roufogalis B Abdolghaffari AH Sahebkar A. Ginger: a complementary approach for management of cardiovascular diseases. Biofactors. (2021) 47:933–51. doi: 10.1002/biof.1777 34388275

[173] Marx W Kiss N Isenring L. Is ginger beneficial for nausea and vomiting? An update of the literature. Curr Opin Support Palliat Care. (2015) 9:189–95. doi: 10.1097/SPC.0000000000000135, PMID: 25872115

[174] Recober A. Pathophysiology of migraine. Continuum (Minneap Minn). (2021) 27:586–96. doi: 10.1212/CON.0000000000000983, PMID: 34048393

[175] Jolad SD Lantz RC Solyom AM Chen GJ Bates RB Timmermann BN. Fresh organically grown ginger (*Zingiber officinale*): composition and effects on LPS-induced PGE2 production. Phytochemistry. (2004) 65:1937–54. doi: 10.1016/j.phytochem.2004.06.008, PMID: 15280001

[176] Ha SK Moon E Ju MS Kim DH Ryu JH Oh MS. 6-Shogaol, a ginger product, modulates neuroinflammation: a new approach to neuroprotection. Neuropharmacology. (2012) 63:211–23. doi: 10.1016/j.neuropharm.2012.03.016 22465818

[177] Maghbooli M Golipour F Moghimi Esfandabadi A Yousefi M. Comparison between the efficacy of ginger and sumatriptan in the ablative treatment of the common migraine. Phytother Res. (2014) 28:412–5. doi: 10.1002/ptr.4996, PMID: 23657930

[178] Martins LB Rodrigues A Rodrigues DF Dos Santos LC Teixeira AL Ferreira AVM. Double-blind placebo-controlled randomized clinical trial of ginger (*Zingiber officinale* Rosc.) addition in migraine acute treatment. Cephalalgia. (2019) 39:68–76. doi: 10.1177/0333102418776016, PMID: 29768938

[179] Chen L Cai Z. The efficacy of ginger for the treatment of migraine: a meta-analysis of randomized controlled studies. Am J Emerg Med. (2021) 46:567–71. doi: 10.1016/j.ajem.2020.11.030, PMID: 33293189

[180] Fiorentini D Cappadone C Farruggia G Prata C. Magnesium: biochemistry, nutrition, detection, and social impact of diseases linked to its deficiency. Nutrients. (2021) 13:1136. doi: 10.3390/nu13041136, PMID: 33808247 PMC8065437

[181] Pelczyńska M Moszak M Bogdański P. The role of magnesium in the pathogenesis of metabolic disorders. Nutrients. (2022) 14:1714. doi: 10.3390/nu14091714, PMID: 35565682 PMC9103223

[182] Barbagallo M Veronese N Dominguez LJ. Magnesium in aging, health and diseases. Nutrients. (2021) 13:463. doi: 10.3390/nu13020463, PMID: 33573164 PMC7912123

[183] Ahmed F Mohammed A. Magnesium: the forgotten electrolyte—a review on hypomagnesemia. Med Sci. (2019) 7:56. doi: 10.3390/medsci7040056, PMID: 30987399 PMC6524065

[184] Botturi A Ciappolino V Delvecchio G Boscutti A Viscardi B Brambilla P. The role and the effect of magnesium in mental disorders: a systematic review. Nutrients. (2020) 12:1661. doi: 10.3390/nu12061661, PMID: 32503201 PMC7352515

[185] Kostov K Halacheva L. Role of magnesium deficiency in promoting atherosclerosis, endothelial dysfunction, and arterial stiffening as risk factors for hypertension. Int J Mol Sci. (2018) 19:1724. doi: 10.3390/ijms19061724, PMID: 29891771 PMC6032400

[186] Domitrz I Cegielska J. Magnesium as an important factor in the pathogenesis and treatment of migraine—from theory to practice. Nutrients. (2022) 14:1089. doi: 10.3390/nu14051089, PMID: 35268064 PMC8912646

[187] Chiu H-Y Yeh T-H Yin-Cheng H Pin-Yuan C. Effects of intravenous and oral magnesium on reducing migraine: a meta-analysis of randomized controlled trials. Pain Physician. (2016) 19:E97–E112. doi: 10.36076/ppj/2016.19.E97, PMID: 26752497

[188] Slavin M Li H Khatri M Frankenfeld C. Dietary magnesium and migraine in adults: a cross-sectional analysis of the National Health and nutrition examination survey 2001–2004. Headache. (2021) 61:276–86. doi: 10.1111/head.14065 33503279

[189] Assarzadegan F Asgarzadeh S Hatamabadi HR Shahrami A Tabatabaey A Asgarzadeh M. Serum concentration of magnesium as an independent risk factor in migraine attacks: a matched case–control study and review of the literature. Int Clin Psychopharmacol. (2016) 31:287–92. doi: 10.1097/YIC.0000000000000130 27140442

[190] Giniatullin R. 5-hydroxytryptamine in migraine: the puzzling role of ionotropic 5-HT3 receptor in the context of established therapeutic effect of metabotropic 5-HT1 subtypes. Br J Pharmacol. (2022) 179:400–15. doi: 10.1111/bph.15710 34643938

[191] de Vries T Villalón CM MaassenVanDenBrink A. Pharmacological treatment of migraine: CGRP and 5-HT beyond the triptans. Pharmacol Ther. (2020) 211:107528. doi: 10.1016/j.pharmthera.2020.107528, PMID: 32173558

[192] Wenwen X Jing Y Yingchao S Qinglu W. The effect of magnesium deficiency on neurological disorders: a narrative review article. Iran J Public Health. (2019) 48:379. 31223564 PMC6570791

[193] Khani S Hejazi SA Yaghoubi M Sharifipour E. Comparative study of magnesium, sodium valproate, and concurrent magnesium-sodium valproate therapy in the prevention of migraine headaches: a randomized controlled double-blind trial. J Headache Pain. (2021) 22:1–10. doi: 10.1186/s10194-021-01234-6 33827421 PMC8028183

[194] Deepika SAK Kumari A Kumar A. Gut brain regulation using psychobiotics for improved neuropsychological illness. Dev Psychobiol. (2023) 65:e22404. doi: 10.1002/dev.22404, PMID: 37338246

[195] Ferrari MD Goadsby PJ Rami B Kurth T Cenk A Charles A. Migraine (Primer). Nat Rev Dis Primers. (2022) 8:2. doi: 10.1038/s41572-021-00328-4 35027572

[196] Crawford J Liu S Tao F. Gut microbiota and migraine. Neurobiol Pain. (2022) 11:100090. doi: 10.1016/j.ynpai.2022.100090, PMID: 35464185 PMC9018445

[197] Aurora SK Shrewsbury SB Ray S Hindiyeh N Nguyen L. A link between gastrointestinal disorders and migraine: insights into the gut–brain connection. Headache. (2021) 61:576–89. doi: 10.1111/head.14099 33793965 PMC8251535

[198] Kim J Lee S Rhew K. Association between gastrointestinal diseases and migraine. Int J Environ Res Public Health. (2022) 19:4018. doi: 10.3390/ijerph19074018, PMID: 35409704 PMC8997650

[199] De Palma G Blennerhassett P Lu J Deng Y Park AJ Green W. Microbiota and host determinants of behavioural phenotype in maternally separated mice. Nat Commun. (2015) 6:7735. doi: 10.1038/ncomms8735 26218677

[200] Aguilera-Lizarraga J Florens M Hussein H Boeckxstaens G. Local immune response as novel disease mechanism underlying abdominal pain in patients with irritable bowel syndrome. Acta Clin Belg. (2022) 77:889–96. doi: 10.1080/17843286.2021.1996069, PMID: 34709996

[201] Todor TS Fukudo S. Systematic review and meta-analysis of calculating degree of comorbidity of irritable bowel syndrome with migraine. Biopsychosoc Med. (2023) 17:22. doi: 10.1186/s13030-023-00275-4, PMID: 37291550 PMC10251688

[202] Sgro M Ray J Foster E Mychasiuk R. Making migraine easier to stomach: the role of the gut-brain-immune axis in headache disorders. Eur J Neurol. (2023) 30:3605–21. doi: 10.1111/ene.15934, PMID: 37329292

[203] Kursun O Yemisci M van den Maagdenberg A Karatas H. Migraine and neuroinflammation: the inflammasome perspective. J Headache Pain. (2021) 22:55. doi: 10.1186/s10194-021-01271-1, PMID: 34112082 PMC8192049

[204] Tamtaji OR Milajerdi A Reiner Ž Asemi Z Dadgostar E Heidari-Soureshjani R. A systematic review and meta-analysis: the effects of probiotic supplementation on metabolic profile in patients with neurological disorders. Compl Therap Med. (2020) 53:102507., PMID: 33066850 10.1016/j.ctim.2020.102507

[205] Stubberud A Buse DC Kristoffersen ES Linde M Tronvik E. Is there a causal relationship between stress and migraine? Current evidence and implications for management. J Headache Pain. (2021) 22:155. doi: 10.1186/s10194-021-01369-6, PMID: 34930118 PMC8685490

[206] Hammond NG Colman I. The role of positive health behaviors in the relationship between early life stress and migraine. Headache. (2020) 60:1111–23. doi: 10.1111/head.13808, PMID: 32320053

[207] Diogenes A Ferraz CC Akopian AN Henry MA Hargreaves KM. LPS sensitizes TRPV1 via activation of TLR4 in trigeminal sensory neurons. J Dent Res. (2011) 90:759–64. doi: 10.1177/0022034511400225, PMID: 21393555

[208] Oroojzadeh P Bostanabad SY Lotfi H. Psychobiotics: the influence of gut microbiota on the gut-brain Axis in neurological disorders. J Mol Neurosci. (2022) 72:1952–64. doi: 10.1007/s12031-022-02053-3, PMID: 35849305 PMC9289355

[209] Cheng LH Liu YW Wu CC Wang S Tsai YC. Psychobiotics in mental health, neurodegenerative and neurodevelopmental disorders. J Food Drug Anal. (2019) 27:632–48. doi: 10.1016/j.jfda.2019.01.002, PMID: 31324280 PMC9307042

[210] Martami F Togha M Seifishahpar M Ghorbani Z Ansari H Karimi T. The effects of a multispecies probiotic supplement on inflammatory markers and episodic and chronic migraine characteristics: a randomized double-blind controlled trial. Cephalalgia. (2019) 39:841–53. doi: 10.1177/0333102418820102, PMID: 30621517

[211] Yong D Lee H Min HG Kim K Oh HS Chu MK. Altered gut microbiota in individuals with episodic and chronic migraine. Sci Rep. (2023) 13:626. doi: 10.1038/s41598-023-27586-4, PMID: 36635330 PMC9835027

[212] Xie Y Zhou G Xu Y He B Wang Y Ma R. Effects of diet based on IgG elimination combined with probiotics on migraine plus irritable bowel syndrome. Pain Res Manag. (2019) 2019:1–6. doi: 10.1155/2019/7890461 PMC6721378 31531150

[213] Ghavami A Khorvash F Heidari Z Khalesi S Askari G. Effect of synbiotic supplementation on migraine characteristics and inflammatory biomarkers in women with migraine: results of a randomized controlled trial. Pharmacol Res. (2021) 169:105668. doi: 10.1016/j.phrs.2021.105668, PMID: 33989763

[214] Parohan M Djalali M Sarraf P Yaghoubi S Seraj A Foroushani AR. Effect of probiotic supplementation on migraine prophylaxis: a systematic review and meta-analysis of randomized controlled trials. Nutr Neurosci. (2022) 25:511–8. doi: 10.1080/1028415X.2020.1764292 32420827

[215] Merenstein D Pot B Leyer G Ouwehand AC Preidis GA Elkins CA. Emerging issues in probiotic safety: 2023 perspectives. Gut Microbes. (2023) 15:2185034. doi: 10.1080/19490976.2023.2185034, PMID: 36919522 PMC10026873

[216] Reyes CM Cornelis MC. Caffeine in the diet: country-level consumption and guidelines. Nutrients. (2018) 10:1772. doi: 10.3390/nu10111772, PMID: 30445721 PMC6266969

[217] Grosso G Godos J Galvano F Giovannucci EL. Coffee, caffeine, and health outcomes: an umbrella review. Annu Rev Nutr. (2017) 37:131–56. doi: 10.1146/annurev-nutr-071816-064941, PMID: 28826374

[218] Guieu R Devaux C Henry H Bechis G Pouget J Mallet D. Adenosine and migraine. Can J Neurol Sci. (1998) 25:55–8. doi: 10.1017/S0317167100033497 9532282

[219] Zaeem Z Zhou L Dilli E. Headaches: a review of the role of dietary factors. Curr Neurol Neurosci Rep. (2016) 16:101. doi: 10.1007/s11910-016-0702-1 27714637

[220] Aurora SK Kori SH Barrodale P McDonald SA Haseley D. Gastric stasis in migraine: more than just a paroxysmal abnormality during a migraine attack. Headache. (2006) 46:57–63. doi: 10.1111/j.1526-4610.2006.00311.x 16412152

[221] Silberstein S. Gastrointestinal manifestations of migraine: meeting the treatment challenges. Headache. (2013) 53:1–3. doi: 10.1111/head.12113, PMID: 23721283

[222] Derry CJ Derry S Moore RA. Caffeine as an analgesic adjuvant for acute pain in adults. Cochrane Database Syst Rev. (2014) 2014:CD009281. doi: 10.1002/14651858.CD009281.pub3 25502052 PMC6485702

[223] Goldstein J Hagen M Gold M. Results of a multicenter, double-blind, randomized, parallel-group, placebo-controlled, single-dose study comparing the fixed combination of acetaminophen, acetylsalicylic acid, and caffeine with ibuprofen for acute treatment of patients with severe migraine. Cephalalgia. (2014) 34:1070–8. doi: 10.1177/0333102414530527, PMID: 24733408

[224] Espiritu AI Del Mundo HC Bico JDP Pasco PMD. The effectiveness and tolerability of oral acetaminophen/aspirin/caffeine (AAC) combination regimen as an acute treatment for migraine in adults: a meta-analysis of randomized trials. Acta Med Philipp. (2017) 51. doi: 10.47895/amp.v50i2.2758

[225] Goldstein J Silberstein SD Saper JR Elkind AH Smith TR Gallagher RM. Acetaminophen, aspirin, and caffeine versus sumatriptan succinate in the early treatment of migraine: results from the ASSET trial. Headache. (2005) 45:973–82. doi: 10.1111/j.1526-4610.2005.05177.x, PMID: 16109110

[226] Peroutka SJ Lyon JA Swarbrick J Lipton RB Kolodner K Goldstein J. Efficacy of diclofenac sodium softgel 100 mg with or without caffeine 100 mg in migraine without aura: a randomized, double-blind, crossover study. Headache. (2004) 44:136–41. doi: 10.1111/j.1526-4610.2004.04029.x, PMID: 14756851

