# Supplementary material for: Practical supplements for prevention and management of migraine attacks: a narrative review
Source: Front Nutr. 2024 Oct 30;11:1433390. doi: 10.3389/fnut.2024.1433390 (PMC11557489; doi:10.3389/fnut.2024.1433390)
Supplement: Supplementary file 1 [file Table_1.pdf]

Supplemental Table 1. The main characteristics of included clinical trial and case-report studies which investigated the effect of nutrient supplements on migraine

First author, year, country (Reference)	Study design	Population	Sample size (Intervention /control group)	Mean age/age range (year)	Intervention (Dosage)	Control (Dosage)	Treatment duration	Main outcomes (↓↑)
Prakash, 2016/ India (1)	Case study	A woman with chronic migraine and Wernicke's Encephalopathy)	1/-	32	Thiamin (500 mg) intravenously, 3 times a day<br><br>Thiamin (200 mg) intravenously, 3 times a day<br><br>Oral thiamin (100 mg), 2 times a day	-	2 days<br><br>2 days<br><br>6 weeks	↓Frequency and intensity of headache<br><br>Improvement in the nystagmus, dizziness, vertigo, and disturbed sleep
		A woman with episodic migraine without aura	1/-	27	Thiamin (500 mg) intravenously, 3 times a day<br><br>Oral thiamin (100 mg), 3 times a day		4 days<br><br>2 months	↓Frequency and intensity of headache<br><br>Improvement in nausea and impaired tandem walk and nystagmus, irritability, vertigo, and disturbed sleep
Antonio, 2018/ Italy (2)	Case study	A man with migraine	1/-	41	Oral thiamine treatment (250 mg/d) started. Every 3 days the dose was increased by 250 mg up to the dose of 750 mg.	-	-	↓Frequency of headache

Schoenen, 1998/ Belgium (3)	RCT	Migraine patients (both genders)	28/26	36.08	Riboflavin (400 mg/d)	Placebo	3 months	↓Attack frequency<br>↓ Migraine days<br>↓ Migraine index, Severity<br>↓ Duration<br>↔ nausea and vomiting
Boehnke, 2004/ Germany (4)	Open label study	Migraine patients (both genders)	23/-	52.09	Riboflavin (400 mg/d)	-	3 and 6 months	↓ Attack frequency<br>↓ Intake of abortive anti-migraine tablets<br>↔ Headache hours and headache intensity
Nambiar, 2011/(5)	Open label trial	Migraine patients (both gender)	50/50	31.5	Riboflavin (100 mg/d)	Propranolol (80mg/day)	3 months	↓Migraine frequency, duration, severity of headache, and migraine disability in both groups<br>↓Side effects in riboflavin group
Rahimdel ,2015/Iran (6)	Single-blind trial	Migraine patients (both genders)	45/45	15-55	Riboflavin (400 mg/d)	Sodium valproate (500 mg/day)	3 months	↓Frequency, median duration per month, and severity of the headaches in both groups<br>↓Side effects in riboflavin group
Velling, 2003/(7)	Case study	A woman with migraine	1/-	62	375 mg of oral sustained-release niacin twice daily for 1 month, and 375 mg once daily for 2 months	-	3 months	↓ Attack frequency
Gedye, 2001/ Canada(8)	Open label clinical trial	Migraine patients (both genders)	12/-	45.4	Tryptophan (500 mg), niacin (100 mg), calcium (500 mg calcium carbonate), caffeine (64 mg),	-	Using for 10 attacks	Migraine relief

					and acetylsalicylic acid (650 mg) and avoid *during a migraine* high-potassium food and magnesium supplements			
Gaby, 2002/(9)	Case study	Female with migraine	1/-	44	Intravenous administration of vitamin C (16 mL), magnesium (5 mL), calcium (4 mL), B6 (2 mL), and 1 mL each of B12, B5, and B complex	-		↓ Attack frequency
Sadeghi, 2015/ Iran (10)	Double-blind trial	Migraine patients (both genders)	26/31	34.24	Pyridoxine (80 mg/d)	Placebo	12 weeks	↓ Headache severity, attacks duration and headache diary results (HDR)<br>↔ Frequency of migraine attacks
Menon, 2012/ Australia (11)	Double-blind trial	Female patients	76/86	44	Folic acid (2mg), vitamin B6 (25mg) and vitamin B12 (400 µg)	Placebo	6 months	↓Homocysteine levels, migraine head pain severity, migraine disability<br>↔Headache frequency
Lea,2008/ Australia (12)	Double-blind trial	Migraine patients (both genders)	37/15	52	Folic acid (2mg), vitamin B6 (25mg) and vitamin B12 (400 µg)	Placebo	6 months	↓Homocysteine levels, migraine disability, Headache frequency and pain severity

Askari, 2017/Iran (13)	Double-blind trial	Migraine patients (both genders)	34/31/30	35.29	Folic acid (5 mg/d) + pyridoxine (80 mg/d)	Placebo	3 months	↓ Headache severity, attack frequency, duration and HDR
					Folic acid alone (5 mg/d)			No significant results
Nematgorgani, 2022/ Iran(14)	Double-blind trial	Female patients	16/16/16/16/ 16/16	36.19	B1 (300 mg/d) B6 (80 mg/d) B9 (2mg/d) B12 (500 μg/d) B complex	Placebo	12 weeks	↓ Use of abortive drugs, frequency of headache, migraine disability in all groups of vitamins
Menon, 2016/ Australia (15)	Double-blind trial	Female patients	126/63	18-60	Combination of Folic acid (1mg), vitamin B6 (25mg) and of vitamin B12 (400 μg)	Placebo	6 months	↔ Migraine Disability, frequency, severity, homocysteine levels
Van der, 2002/ Netherlands (16)	Open label study	Migraine patients (both genders)	19/-	41	Intranasal OHB12 (1 mg/d)	-	3 months	↓ Frequency and duration of the migraine attacks, migraine days per month, amount of acute migraine medication ↔Other symptoms
Visser, 2020/ Australia(17)	Double blind trial	Migraine patients (both genders)	19/16	44.7	N-acetylcysteine (1200 mg), vitamin E (500 IU), and vitamin C (1000 mg) daily	Placebo	3 months	↓ Frequency, severity and duration of headache, and acute medication use
Chayasirisobhon, 2006/ Los Angeles (18)	Open label study	Migraine patients (both genders)	11/-	41.1	Pine bark extract (120 mg), vitamin C (60 mg), and vitamin E daily (30 IU)	-	3 months	↓ Frequency and severity of headache, migraine disability

Chayasirisobhon, 2013/ U.S.A(19)	Open clinical trial	Migraine patients (both genders)	50/-	39.46	Pinus radiata bark extract (1200 mg) and vitamin C daily (150 mg)	-	3 months	↓ Frequency, severity and disability of headache
Mottaghi, 2015/ Iran (20)	Double blind trial	Migraine patients (both genders)	33/32	33.3	Vitamin D (50,000 IU/week)	Placebo	10 weeks	↓Migraine frequency,<br>↓ HDR<br><br>↔ Severity and duration of headache attacks
Susanti, 2023/ Indonesia (21)	Trial	Migraine patients (both genders)	12/12	33.9	Vitamin D (2000 IU/day)	Placebo	12 weeks	↓ Frequency and duration of migraine attacks<br>↓ Glutamate and NLRP3<br>↔ Plasma CGRP levels<br>↑ Vitamin D3 serum
Ghorbani, 2020/ Iran (22)	Double blind trial	Migraine patients (both gender)	38/36	37.5	Vitamin D 2000 IU (50 µg)	Placebo	12 weeks	↓Migraine frequency, duration, severity of headache<br>↓ Analgesics use/month<br>↓ iNOS, IL-6<br>↔ IL-10 and Cox-2
Ziaei, 2009/ Iran (23)	Cross-over	Women with menstrual migraine	72	-	Vitamin E (400 IU daily) for five days	Placebo	Five days, (two days before to three days after menstruation for two cycles followed by a one-month wash-out)	↓ Migraine severity and functional disability scales, photophobia, phonophobia, and nausea

Pradalier, 2001/France (24)	Double-blind trial	Migraine patients (both genders)	96/87	39	Omega-3 PUFA (6 g/d)	Placebo	4 months	↔ Mean duration and intensity of the migraine attacks, rescue medication use<br>↓Total number of attacks
Tajmirriahi, 2012/Iran (25)	Single-blind trial	Migraine patients (both genders)	29/38	35.3	Fish oil (180 mg/d) + Sodium valproate (400 mg/d)	Sodium valproate (400 mg/d)	12 weeks	↓Duration, monthly frequency, and severity of headache in both group<br>↓ Monthly frequency, and severity of headache in intervention compare to control group
Wang, 2024/ Taiwan(26)	Double-blind trial	Migraine patients (both genders)	35/35	39.4	2 g fish oil containing 1.8 g of EPA	Placebo (2 g soybean oil daily)	12 weeks	↓ Migraine frequency, days using acute headache medication, headache severity, migraine disability, anxiety and depression, and quality of life
Ramsden, 2013/ USA(27)	Single-blind trial	Migraine patients (both genders)	33/34	41.5	A diet with high n-3 plus low n-6 fatty acids	Diet with low n-6 fatty acids	12 weeks	↓ Headache impact test, number of headache days per month, headache hours per day, n-6 in HUFA score<br>↑ Antinociceptive n-3 pathway markers 18-hydroxy eicosapentaenoic acid and 17-hydroxy-docosahexaenoic acid
Djalali, 2022/Iran (28)	Double-blind trial	Migraine patients	20/20	-	EPA (600 mg) and DHA (300 mg), twice daily	Placebo	2 months	↑ Anti-inflammatory cytokine IL-4<br>↓ Pro-inflammatory cytokine IFN-γ<br>↔ Serum TGF-β and IL-17 levels

Rezaei Kelishadi, 2021/ Iran (29)	Double blind trial	Women with episodic migraine	42/37	41.7	Alpha-lipoic acid (300 mg), twice a day	Placebo	3 months	↓ MDA, CRP, depression, anxiety, and stress<br>↔ GSH, TAC, TOS
Cavestro, 2017/ Italy (30)	Open-label exploratory study of a cohort study	Migraine patients with insulin resistance	32	44	Alpha-lipoic acid (400 mg/day)	-	6 months	↓ Number of attacks and days of treatment in migraine patients with with insulin resistance<br>↔ Metabolic indices and insulin sensitivity markers
Magis, 2006/ Belgium (31)	Double blind trial	Migraine patients (both genders)	26/18	38	Thioctic acid (α-lipoic acid) 600 mg/day	Placebo	3 months	↓ Attack frequency, headache days, and headache severity
Dahri, 2019/ Iran (32)	Double blind trial	Non-menopausal Women with migraine	23/22	32.3	Coenzyme Q10 (400 mg/d)	Placebo (Wheat starch)	3 months	↓ Frequency, duration, and severity of headache<br>↓ CGRP and TNF-α<br>↑ serum CoQ10 levels<br>↔ IL-6 and IL-10
Shoeibi, 2016/ Iran (33)	Open-label trial	Migraine patients (both genders)	36/37	33.5	Preventive drugs + coenzyme Q10 (100 mg/d)	Preventive drugs	3 months	↓ Frequency, duration, and severity of headache<br>↓ Nausea, photophobia, phonophobia<br>↔ Vomiting
Dahri, 2023/Iran (34)	Double blind trial	Migraine patients (both genders)	39/38	33.7	Coenzyme Q10 (400 mg/d)	Placebo	12 weeks	↓MDA, BFP<br>↔TAC, LDL, TG, Tcho<br>↑Serum Co-Q10, HDL-C

Hajihashemi, 2018/ Iran (35)	Double blind trial	Migraine patients (both genders)	24/26	32.4	Coenzyme Q10 (30 mg/d) and L-carnitine (500 mg/d)	Placebo	8 weeks	↓ Frequency, duration, and severity of headache, HDR, Lactate
Taha, 2023/Iraq (36)	Trial	Migraine patients (both genders)	40/40	30.49	Coenzyme Q10 (400 mg/d) in combination with other prophylactic antimigraine drugs	Coenzyme Q10 (400 mg/d)	3 months	↓ Frequency, duration of headache, migraine disability, and symptoms such as vomiting and sound sensitivity
Maghbooli, 2013/ Iran (37)	Double blind trial	Migraine patients	50/50	34.5	Ginger powder (250 mg/d)	Sumatriptan (50 mg/d)	1 month	↓ Severity of headaches in both groups<br>Less side effects in ginger group
Martins, 2018/ (38)	Double blind trial	Migraine patients (both genders)	30/30	32.5	Ginger extract (400 mg/d) + ketoprofen (100 mg)	Placebo + ketoprofen (100 mg)	Acute treatment while migraine attack	↓Pain<br>↑Improvement on functional status<br>Better clinical response after 1, 1.5, and 2 hours in ginger extract group
Khani, 2021/ Iran (39)	Double blind trial	Migraine patients (both genders)	82/70/70	35.5	Sodium Valproate (200 mg) + Magnesium (250 mg) twice daily<br><br>Magnesium (250 mg) + Placebo twice daily	Placebo + Sodium Valproate (200 mg) twice a day	12 weeks	↑ Antimigraine properties of sodium valproate in combination with magnesium<br>↓ Valproate dose for migraine prophylaxis<br>↓ frequency, intensity, duration of attacks, number of medications taken, and migraine disability in all groups, but the combination of magnesium and sodium valproate was more effective

Martami, 2019/ Iran (40)	Double blind trial	Episodic and chronic migraine patients	21,22/18,18	38.07	14-strain probiotic mixture, 2 capsules/day	Placebo	10 weeks in the episodic migraine group<br><br>8 weeks in the chronic migraine group	↓ Attack frequency, severity, number of migraine days, number of abortive drugs in both episodic and chronic migraine patients<br>↓ Migraine attack duration in chronic migraine patients<br>↔ Inflammatory markers
Xie, 2019/ China (41)	trial	Migraine patients with IBS (both genders)	20/20/20	-	A (IgG elimination diet combined with probiotics)<br><br>B (probiotics)	C (IgG eliminatio n diet)	14 weeks	↓ Migraine days (in the groups of A and C)<br>↓Use of over-the counter analgesics and disability (in all groups)<br>↑ Serum serotonin (in the groups of A and C)<br>Improvement in bowel habits (in all groups)
Ghavami, 2021 Iran (42)	Double blind trial	Women with migraine	40/40	37.83	Synbiotic supplement (12 probiotic strains + fructooligosacchar ides prebiotic) Twice a day	Placebo	12 weeks	↓ Migraine attacks frequency<br>↓Number of painkillers used<br>↓ Gastrointestinal problems<br>↓ Zonulin and Hs-CRP levels<br>↔ Migraine severity and duration
Goldstein, 2014/ USA (43)	Double blind trial	Migraine patients (both genders)	286/291/83	38.2	Combination of acetaminophen (500 mg), acetylsalicylic acid (500 mg), and caffeine (130 mg) daily (ACC)	Ibuprofen (400 mg)<br><br>Placebo	Acute treatment while migraine attack	↑Pain relief, headache response<br>↓Functional disability, pain intensity, phonophobia, photophobia, use of rescue medication<br>(Although these items were significant for both ACC and Ibuprofen, the efficacy of

								ACC was better than Ibuprofen)
Goldstein, 2005/ (44)	Double blind trial	Migraine patients (both gender)	69/67/35	38.1	Acetaminophen (500 mg), aspirin (500 mg), and caffeine (130 mg) daily	Sumatriptan (50 mg)<br><br>Placebo	Acute treatment while migraine attack	↓ Pain intensity, Pain, associated symptoms, use of rescue medication, disability ↑Response,
Peroutka, 2004/San Francisco (45)	Randomized, Double-blind, Crossover	Migraine patients (both gender)	72	44.7	Diclofenac sodium (100 mg) soft gel + caffeine (100 mg)	Diclofenac sodium (100 mg) soft gel<br><br>Placebo	Acute treatment while migraine attack	↓ Headache, Rescue medication

Abbreviations: TNF-α, tumor necrosis factor-α; CGRP, calcitonin gene related peptide; COX-2, cyclooxygenase-2; iNOS, inducible nitric oxide synthase; HDR, headache diary results; OHB12, Hydroxocobalamin; IL-6, interleukin 6; hs-CRP, high-sensitivity C-reactive protein; RCT, randomized clinical trial; PUFA, poly unsaturated fatty acids; EPA, eicosapentaenoic acid; DHA, docosahexaenoic acid; MDA, malondialdehyde; CRP, C-reactive protein; CoQ10, Coenzyme Q10; AAC, combination of acetaminophen 500 mg, acetylsalicylic acid 500 mg, and caffeine 130 mg; NLRP3; NOD-Like Receptor Protein 3, BFP, body fat percent; HDL-c, high density lipoprotein-cholesterol; IL-4, interleukin-4; IFN-γ, interferon-gamma; TGF-β, Transforming growth factor β; GSH, Glutathione; TAC, total antioxidant capacity; TOS, total oxidant capacity; LDL-c, low density lipoprotein-cholesterol; Tg, triglyceride; Tcho, Total cholesterol.

REFERENCES

1. Prakash S, Kumar Singh A, Rathore C. Chronic Migraine Responding to Intravenous Thiamine: A Report of Two Cases. Headache. 2016;56(7):1204-9.
2. Antonio C, Massimo T, Gianpaolo Z, Immacolata PM, Erika T. Oral High-Dose Thiamine Improves the Symptoms of Chronic Cluster Headache. Case Rep Neurol Med. 2018;2018:3901619.
3. Schoenen J, Jacquy J, Lenaerts M. Effectiveness of high-dose riboflavin in migraine prophylaxis A randomized controlled trial. Neurology. 1998;50(2):466-70.
4. Boehnke C, Reuter U, Flach U, Schuh-Hofer S, Einhäupl K, Arnold G. High-dose riboflavin treatment is efficacious in migraine prophylaxis: an open study in a tertiary care centre. European Journal of Neurology. 2004;11(7):475-7.
5. Nambiar N, Aiyappa C, Srinivasa R. Oral riboflavin versus oral propranolol in migraine prophylaxis: An open label randomized controlled trial. Neurology Asia. 2011;16(3).
6. Rahimdel A, Zeinali A, Yazdian-Anari P, Hajizadeh R, Arefnia E. Effectiveness of vitamin B2 versus sodium valproate in migraine prophylaxis: a randomized clinical trial. Electronic Physician. 2015;7(6):1344.
7. Velling DA, Dodick DW, Muir JJ. Sustained-release niacin for prevention of migraine headache. Mayo Clin Proc. 2003;78(6):770-1.
8. Gedye A. Hypothesized treatment for migraines using low doses of tryptophan, niacin, calcium, caffeine, and acetylsalicylic acid. Med Hypotheses. 2001;56(1):91-4.
9. Gaby AR. Intravenous nutrient therapy: the "Myers' cocktail". Altern Med Rev. 2002;7(5):389-403.
10. Sadeghi O, Nasiri M, Maghsoudi Z, Pahlavani N, Rezaie M, Askari G. Effects of pyridoxine supplementation on severity, frequency and duration of migraine attacks in migraine patients with aura: A double-blind randomized clinical trial study in Iran. Iranian journal of neurology. 2015;14(2):74.
11. Menon S, Lea RA, Roy B, Hanna M, Wee S, Haupt LM, et al. Genotypes of the MTHFR C677T and MTRR A66G genes act independently to reduce migraine disability in response to vitamin supplementation. Pharmacogenetics and genomics. 2012;22(10):741-9.
12. Lea R, Colson N, Quinlan S, Macmillan J, Griffiths L. The effects of vitamin supplementation and MTHFR (C677T) genotype on homocysteine-lowering and migraine disability. Pharmacogenetics and genomics. 2009;19(6):422-8.
13. Askari G, Nasiri M, Mozaffari-Khosravi H, Rezaie M, Bagheri-Bidakhavidi M, Sadeghi O. The effects of folic acid and pyridoxine supplementation on characteristics of migraine attacks in migraine patients with aura: A double-blind, randomized placebo-controlled, clinical trial. Nutrition. 2017;38:74-9.
14. Nematgorgani S, Razeghi-Jahromi S, Jafari E, Togha M, Rafiee P, Ghorbani Z, et al. B vitamins and their combination could reduce migraine headaches: A randomized double-blind controlled trial. Current Journal of Neurology. 2022;21(2):105.
15. Menon S, Nasir B, Avgan N, Ghassabian S, Oliver C, Lea R, et al. The effect of 1 mg folic acid supplementation on clinical outcomes in female migraine with aura patients. The journal of headache and pain. 2016;17(1):1-7.
16. Van der Kuy P-H, Merkus F, Lohman J, Berg Jt, Hooymans P. Hydroxocobalamin, a nitric oxide scavenger, in the prophylaxis of migraine: an open, pilot study. Cephalalgia. 2002;22(7):513-9.

17. Visser EJ, Drummond PD, Lee-Visser JLA. Reduction in Migraine and Headache Frequency and Intensity With Combined Antioxidant Prophylaxis (N-acetylcysteine, Vitamin E, and Vitamin C): A Randomized Sham-Controlled Pilot Study. Pain Pract. 2020;20(7):737-47.
18. Chayasirisobhon S. Use of a pine bark extract and antioxidant vitamin combination product as therapy for migraine in patients refractory to pharmacologic medication. Headache. 2006;46(5):788-93.
19. Chayasirisobhon S. Efficacy of Pinus radiata bark extract and vitamin C combination product as a prophylactic therapy for recalcitrant migraine and long-term results. Acta Neurol Taiwan. 2013;22(1):13-21.
20. Mottaghi T, Askari G, Khorvash F, Maracy MR. Effect of Vitamin D supplementation on symptoms and C-reactive protein in migraine patients. Journal of research in medical sciences: the official journal of Isfahan University of Medical Sciences. 2015;20(5):477.
21. Susanti R, Syafrita Y, Rita RS, Darwin E, Lipoeto NI, Ali H, et al. The Influence of Vitamin D3 Administration on the Levels of CGRP, Glutamate, and NLRP3 during the Ictal Phase in Chronic Migraine Patients. Pharmacognosy Journal. 2023;15(6).
22. Ghorbani Z, Togha M, Rafiee P, Ahmadi ZS, Rasekh Magham R, Djalali M, et al. Vitamin D3 might improve headache characteristics and protect against inflammation in migraine: a randomized clinical trial. Neurological Sciences. 2020;41:1183-92.
23. Ziaei S, Kazemnejad A, Sedighi A. The effect of vitamin E on the treatment of menstrual migraine. Medical science monitor: international medical journal of experimental and clinical research. 2009;15(1):CR16-9.
24. Pradalier A, Bakouche P, Baudesson G, Delage A, Cornaille-Lafage G, Launay J, et al. Failure of omega-3 polyunsaturated fatty acids in prevention of migraine: a double-blind study versus placebo. Cephalalgia. 2001;21(8):818-22.
25. Tajmirriahi M, Sohelipour M, Basiri K, Shaygannejad V, Ghorbani A, Saadatnia M. The effects of sodium valproate with fish oil supplementation or alone in migraine prevention: A randomized single-blind clinical trial. Iranian journal of neurology. 2012;11(1):21.
26. Wang H-F, Liu W-C, Zailani H, Yang C-C, Chen T-B, Chang C-M, et al. A 12-week randomized double-blind clinical trial of eicosapentaenoic acid intervention in episodic migraine. Brain, Behavior, and Immunity. 2024;118:459-67.
27. Ramsden CE, Faurot KR, Zamora D, Suchindran CM, MacIntosh BA, Gaylord S, et al. Targeted alteration of dietary n-3 and n-6 fatty acids for the treatment of chronic headaches: a randomized trial. PAIN®. 2013;154(11):2441-51.
28. Djalali M, Talebi S, Djalali E, Abdolahi M, Travica N, Djalali M. The effect of omega-3 fatty acids supplementation on inflammatory biomarkers in subjects with migraine: a randomized, double-blind, placebo-controlled trial. Immunopharmacology and Immunotoxicology. 2023:1-6.
29. Rezaei Kelishadi M, Alavi Naeini A, Askari G, Khorvash F, Heidari Z. The efficacy of alpha-lipoic acid in improving oxidative, inflammatory, and mood status in women with episodic migraine in a randomised, double-blind, placebo-controlled clinical trial. International Journal of Clinical Practice. 2021;75(9):e14455.
30. Cavestro C, Bedogni G, Molinari F, Mandrino S, Rota E, Frigeri MC. Alpha-lipoic acid shows promise to improve migraine in patients with insulin resistance: a 6-month exploratory study. Journal of medicinal food. 2018;21(3):269-73.
31. Magis D, Ambrosini A, Sándor P, Jacquy J, Laloux P, Schoenen J. A randomized double-blind placebo-controlled trial of thioctic acid in migraine prophylaxis. Headache: The Journal of Head and Face Pain. 2007;47(1):52-7.
32. Dahri M, Tarighat-Esfanjani A, Asghari-Jafarabadi M, Hashemilar M. Oral coenzyme Q10 supplementation in patients with migraine: Effects on clinical features and inflammatory markers. Nutr Neurosci. 2019;22(9):607-15.

33. Shoeibi A, Olfati N, Soltani Sabi M, Salehi M, Mali S, Akbari Oryani M. Effectiveness of coenzyme Q10 in prophylactic treatment of migraine headache: an open-label, add-on, controlled trial. Acta Neurol Belg. 2017;117(1):103-9.
34. Dahri M, Sadeghi AS, Pahlavani N, Nattagh-Eshtivani E, Hashemilar M, Asghari-Jafarabadi M, et al. The effects of coenzyme Q10 supplementation on oxidative status and lipid profile in migraine patients: a randomized double-blinded controlled clinical trial. Clinical nutrition research. 2023;12(4):257.
35. Hajihashemi P, Askari G, Khorvash F, Reza Maracy M, Nourian M. The effects of concurrent Coenzyme Q10, L-carnitine supplementation in migraine prophylaxis: A randomized, placebo-controlled, double-blind trial. Cephalalgia. 2019;39(5):648-54.
36. Taha M, Abdulwahhab M, Mostafa A. The Effect of Coenzyme Q10 as a Prophylactic Treatment in Episodic Migraine. Duzce Medical Journal. 2023;25(2):147-51.
37. Maghbooli M, Golipour F, Moghimi Esfandabadi A, Yousefi M. Comparison between the efficacy of ginger and sumatriptan in the ablative treatment of the common migraine. Phytother Res. 2014;28(3):412-5.
38. Martins LB, Rodrigues A, Rodrigues DF, Dos Santos LC, Teixeira AL, Ferreira AVM. Double-blind placebo-controlled randomized clinical trial of ginger (Zingiber officinale Rosc.) addition in migraine acute treatment. Cephalalgia. 2019;39(1):68-76.
39. Khani S, Hejazi SA, Yaghoubi M, Sharifipour E. Comparative study of magnesium, sodium valproate, and concurrent magnesium-sodium valproate therapy in the prevention of migraine headaches: a randomized controlled double-blind trial. The Journal of Headache and Pain. 2021;22(1):1-10.
40. Martami F, Togha M, Seifishahpar M, Ghorbani Z, Ansari H, Karimi T, et al. The effects of a multispecies probiotic supplement on inflammatory markers and episodic and chronic migraine characteristics: A randomized double-blind controlled trial. Cephalalgia. 2019;39(7):841-53.
41. Xie Y, Zhou G, Xu Y, He B, Wang Y, Ma R, et al. Effects of Diet Based on IgG Elimination Combined with Probiotics on Migraine Plus Irritable Bowel Syndrome. Pain research & management. 2019;2019:7890461.
42. Ghavami A, Khorvash F, Heidari Z, Khalesi S, Askari G. Effect of synbiotic supplementation on migraine characteristics and inflammatory biomarkers in women with migraine: Results of a randomized controlled trial. Pharmacological research. 2021;169:105668.
43. Goldstein J, Hagen M, Gold M. Results of a multicenter, double-blind, randomized, parallel-group, placebo-controlled, single-dose study comparing the fixed combination of acetaminophen, acetylsalicylic acid, and caffeine with ibuprofen for acute treatment of patients with severe migraine. Cephalalgia. 2014;34(13):1070-8.
44. Goldstein J, Silberstein SD, Saper JR, Elkind AH, Smith TR, Gallagher RM, et al. Acetaminophen, aspirin, and caffeine versus sumatriptan succinate in the early treatment of migraine: results from the ASSET trial. Headache. 2005;45(8):973-82.
45. Peroutka SJ, Lyon JA, Swarbrick J, Lipton RB, Kolodner K, Goldstein J. Efficacy of diclofenac sodium softgel 100 mg with or without caffeine 100 mg in migraine without aura: a randomized, double-blind, crossover study. Headache. 2004;44(2):136-41.
